# Totally laparoscopic versus laparoscopy-assisted distal gastrectomy: the KLASS-07: a randomized controlled trial

**DOI:** 10.1097/JS9.0000000000001543

**Published:** 2024-05-06

**Authors:** Shin-Hoo Park, Chang-Min Lee, Hoon Hur, Jae-Seok Min, Seung Wan Ryu, Young-Gil Son, Hyun Dong Chae, Oh Jeong, Mi Ran Jung, Chang In Choi, Kyo Young Song, Han Hong Lee, Ho Goon Kim, Ye Seob Jee, Sun-Hwi Hwang, Moon-Soo Lee, Kwang Hee Kim, Sang Hyuk Seo, In Ho Jeong, Myoung Won Son, Chang Hyun Kim, Moon-Won Yoo, Sung Jin Oh, Jeong Goo Kim, Seong Ho Hwang, Sung IL Choi, Kyung Sook Yang, Hua Huang, Sungsoo Park

**Affiliations:** a Department of Surgery, Korea University College of Medicine; b Department of Biostatistics, Korea University College of Medicine; c Division of Foregut Surgery, Korea University Anam Hospital; d Department of Surgery, Seoul St Mary’s Hospital; e Department of Surgery, Incheon St. Mary’s Hospital; f Department of Surgery, Daejeon St. Mary’s Hospital, College of Medicine, The Catholic University of Korea, Seoul; g Department of Surgery, Asan Medical Centre, University of Ulsan College of Medicine; h Department of Surgery, Kyung Hee University Hospital at Gangdong, Seoul; i Department of Surgery, Uijeongbu Eulji Medical Centre, Eulji University College of Medicine; j Department of Surgery, Eulji University Hospital, Daejeon; k Department of Surgery, Korea University Ansan Hospital, Ansan; l Department of Surgery, Ajou University School of Medicine, Suwon; m Department of Surgery, Dongnam Institute of Radiological and Medical Sciences, Cancer Centre; n Department of Surgery, Haeundae Paik Hospital, Inje University College of Medicine, Busan; o Department of Surgery, Keimyung University Dongsan Medical Centre; p Department of Surgery, Catholic University of Daegu School of Medicine, Daegu; q Department of Surgery, Chonnam National University Medical School, Jeollanam-do; r Department of Surgery, Pusan National University School of Medicine, Pusan; s Department of Surgery, Chonnam National University Medical School, Gwangju; t Department of Surgery, Dankook University College of Medicine, Cheonan; u Pusan National University School of Medicine, Research Institute for Convergence of Biomedical Science and Technology, Department of Surgery, Pusan National University Yangsan Hospital, Yangsan; v Department of Surgery, Busan Paik Hospital, Inje University, Gimhae; w Department of Surgery, Jeju National University School of Medicine, Jeju; x Department of Surgery, Soonchunhyang University Hospital Cheonan, Cheonan, Korea; y Department of Gastric Surgery, Fudan University Shanghai Cancer Centre; z Department of Oncology, Shanghai Medical College, Fudan University, Shanghai, China

**Keywords:** clinical trial, gastrectomy, gastric cancer, laparoscopic surgery, morbidity, phase III, quality of life

## Abstract

**Backgrounds:**

Strong evidence is lacking as no confirmatory randomized controlled trials (RCTs) have compared the efficacy of totally laparoscopic distal gastrectomy (TLDG) with laparoscopy-assisted distal gastrectomy (LADG). The authors performed an RCT to confirm if TLDG is different from LADG.

**Methods:**

The KLASS-07 trial is a multi-centre, open-label, parallel-group, phase III, RCT of 442 patients with clinical stage I gastric cancer. Patients were enroled from 21 cancer care centres in South Korea between January 2018 and September 2020 and randomized to undergo TLDG or LADG using blocked randomization with a 1:1 allocation ratio, stratified by the participating investigators. Patients were treated through R0 resections by TLDG or LADG as the full analysis set of the KLASS-07 trial. The primary endpoint was morbidity within postoperative day 30, and the secondary endpoint was quality of life (QoL) for 1 year. This trial is registered at ClinicalTrials.gov (NCT 03393182).

**Results:**

Four hundred forty-two patients were randomized (222 to TLDG, 220 to LADG), and 422 patients were included in the pure analysis (213 and 209, respectively). The overall complication rate did not differ between the two groups (TLDG vs. LADG: 12.2% vs. 17.2%). However, TLDG provided less postoperative ileus and pulmonary complications than LADG (0.9% vs. 5.7%, *P=*0.006; and 0.5% vs. 4.3%, *P=*0.035, respectively). The QoL was better after TLDG than after LADG regarding emotional functioning at 6 months, pain at 3 months, anxiety at 3 and 6 months, and body image at 3 and 6 months (all *P<*0.05). However, these QoL differences were resolved at 1 year.

**Conclusions:**

The KLASS-07 trial confirmed that TLDG is not different from LADG in terms of postoperative complications but has the advantages to reduce ileus and pulmonary complications. TLDG can be a good option to offer better QoL in terms of pain, body image, emotion, and anxiety at 3–6 months.

**Figure:**
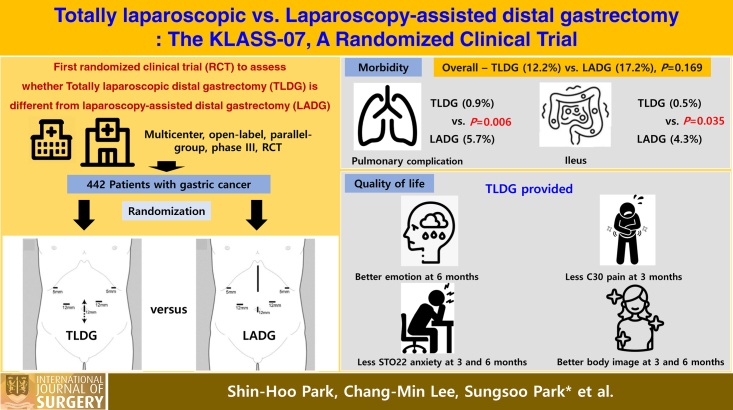

## Introduction

Highlights

- The KLASS-07-randomized controlled trial (RCT) proved that totally laparoscopic distal gastrectomy (TLDG) is not different from laparoscopy-assisted distal gastrectomy (LADG) in terms of postoperative morbidities within 30 days (TLDG vs. LADG: 12.2% vs. 17.2%) when used for clinical stage I gastric cancer treatment but has benefits in terms of reducing ileus (0.9% vs. 5.7%, *P*=0.006) and pulmonary complications (0.9% vs. 4.3%, *P*=0.035).
- TLDG can be a good option to offer better quality of life (QoL) in terms of pain, body image, emotions, and anxiety at 3 or 6 months, but these QoL differences resolved by 1 year postoperatively.
- The results of this study show that TLDG is a safe and effective treatment in patients with early gastric cancer such as LADG. Although, the current gastric cancer treatment guidelines states that the laparoscopic approach as a treatment option for stage I gastric cancer, the relevant evidence is based on outcomes after laparoscopy-assisted surgery.
- Based on the results of this study, it seems that TLDG can also be described as a treatment option for stage I gastric cancer in the future. The decision to use TLDG or LADG depends on the qualified surgeon, as these QOL differences usually resolve by 1 year postoperatively.
- In addition, future interest will be focused on whether reduced port or single-incisional gastrectomy can provide even less invasiveness compared to TLDG or LADG.

Curative gastric cancer surgery has long been performed through the epigastric incision for natural anatomical access. Although the safety and benefits of laparoscopy-assisted distal gastrectomy (LADG) have been verified, it remains unchanging that an additional epigastric incision is still required for gastric resection and gastrointestinal reconstruction^1–8^. High interest in minimally invasive surgery and improvements in laparoscopic surgical techniques have eventually led to the emergence of totally laparoscopic distal gastrectomy (TLDG), in which all procedures are performed intracorporeally without the need for an epigastric incision. However, in the current era of laparoscopic surgery, surgeons’ preferences, vague expectations for minimal invasiveness, technical issues, and concerns for postoperative outcomes have led to the coexistence of TLDG and LADG.

While current gastric cancer treatment guidelines state that LADG can be considered an option to treat clinical stage I cancer, no firm consensus has yet been established on TLDG as a treatment option or its difference over LADG^9,10^. TLDG has been reported to be favourable in terms of fewer wound complications and easier anastomosis regardless of tumour location or patient obesity^11–13^. A pre-study survey of this RCT using meta-analysis reported that TLDG was favourable for less blood loss, analgesic use, and earlier recovery^14^. However, Choi *et al*.^15^ reported that patients with LADG had shorter time-to-first meals and hospital stays than those with TLDG. Despite numerous retrospective studies, they have rarely focused on quality of life (QoL), which reflects the impact of intervention from the patient’s stance of view for a considerable time after surgery. Considering that the changes in gastrointestinal physiology-related QoL should be assessed for at least 1 year, a previous single-centre clinical trial analyzing QoL only at 3 months cannot represent the general outcome; thus, their results can be classified as level II evidence^16–19^.

To acquire stronger level I evidence, the we conducted a prospective randomised controlled trial (RCT) to compare the results of TLDG and LADG in terms of postoperative morbidities within 30 days and QoL for patients with clinical stage I gastric cancer.

## METHODS

### Study design and participants

KLASS-07-RCT was an open-label, parallel-group, multi-centre, investigator-initiated, phase III, prospective RCT conducted by 26 investigators from 21 tertiary teaching hospitals in South Korea. The trial’s preliminary study and protocol were published before and registered with ClinicalTrials. gov (NCT number: 03393182)^20,21^. This study was approved by the Institutional Review Board (IRB) of the Korea University Medical Centre (No. 2017AN0328) and each participating institution. The work has been reported in line with Consolidated Standards of Reporting Trials (CONSORT) Guidelines^22^, Supplemental Digital Content 1, http://links.lww.com/JS9/C488.

Inclusion criteria were as follows: (1) age 20–80 years; (2) Eastern Cooperative Oncology Group performance status score of 0 or 1; (3) histologically proven clinical stage I gastric cancer; and (4) written informed consent for participation. We excluded patients with (1) a history of previous gastric resection or major abdominal surgeries, (2) a need for combined resection because of other primary malignancies, (3) experiences of chemo- or radiotherapy within the last 5 years, and (4) cognitive impairment, pregnancy, and participation in another trial within the last 6 months^21^.

### End points

The objective of the trial was to verify whether TLDG is different from LADG in clinical stage I gastric adenocarcinoma. The primary endpoint was early postoperative morbidity within 30 days after surgery. And, morbidities were categorized according to local or systemic complications, and their severity was graded using the Clavien–Dindo classification system^23^. The secondary endpoint was QoL within 1 year after surgery, assessed using the Korean version of the European Organization for Research and Treatment of Cancer (EORTC) QLQ-C30 (version 3.0) and STO22 questionnaires^24^. Other end points included short-term outcomes, late morbidities after postoperative day 30, nutritional status, and gastroscopic findings at 6 and 12 months postoperatively.

### Randomisation, masking and data management

After informed consent was obtained, we registered each patient in the trial by filling in the screening data such as baseline demographics, into an electronic recording system (available at https://www.klass07.com). Then, a web-based, centralized, independent registration system provided an allocation code number (SAS software, version 9.2), and patients were allocated in the sequence of date of enrolment. To minimize the bias resulting from differences in surgeons’ technical experiences and proficiency, a blocked randomisation design in a 1:1 allocation ratio, stratified by participating investigators, was used. The block size was confidential and did not allow investigators to maintain the randomisation properties. An independent monitoring and steering committee supervised the progress and safety of the trial. Investigators noticed the randomisation code via e-mail, and surgeons immediately gave information to the patients regarding the type of operation they would undergo to fulfil patients’ “right to know,” according to the IRB’s ethical recommendation. Finally, the research coordinators collected all data from each hospital.

### Surgical interventions and quality control

After exploring the peritoneal cavity, a conventional distal gastrectomy with lymphadenectomy, including partial omentectomy, was performed either by a totally laparoscopic or laparoscopy-assisted method^9,10^. Lymph node dissection at station 14v was optional. In the TLDG, all procedures were performed intracorporeally. Mini-laparotomy, extending from the umbilical incision, was performed only to retrieve the resected stomach. During LADG, a mini-laparotomy was performed near the epigastrium in the upper abdomen. Through mini-laparotomy, gastric division, and gastrointestinal reconstruction were extracorporeally performed. Gastrojejunostomy was conducted using the standard Billroth II, Billroth II with Braun anastomosis, Roux-en-Y, or uncut Roux-en-Y reconstruction, according to the surgeon’s preference. During the reconstructive procedures, linear stapling or hand-sewing techniques were used to close the common entry hole after connecting the stomach and jejunum or the jejunum and jejunum with a linear stapler. All mesenteric defects were closed. As described in the protocol^21^, Billroth I (BI) reconstruction was not performed in this trial because it was regarded as a confounder^21,25^.

To accurately assess blood loss, irrigation was not permitted during surgery, except in cases of serious bleeding. We defined the “Conversion to open” as an additional abdominal incision for hand-assisted procedures before completing the laparoscopic lymphadenectomy.

The detailed criteria for participating surgeons in KLASS-07-RCT have been described previously^21^. To participate in the trial, the surgeons had to meet the following criteria: surgeons had performed at least 50 gastrectomies (≥25 cases of each TLDG and LADG) and over 30 gastrectomies annually. Before the initiation of the trial, we reported a multi-centre, preliminary study that showed no significant differences in postoperative results between TLDG and LADG and set up a standardized protocol for every surgical procedure^20^. All participating investigators and regular steering committees reviewed each other’s unrefined videos and, if needed, provided technical feedback to make a consensus on procedural standardization. During operation, videotaping of the laparoscopic procedures and documentary photography of the operative field after lymphatic dissection were obligatory. In addition, some investigators had already participated in previous KLASS trials.

#### Perioperative care and follow-up

Postoperative care and discharge criteria were similar across the participating institutions following a standardized clinical pathway. The diet began from permitting water to a semi-fluid and a soft/bland diet depending on the patient’s status. When the patients were stable 2–3 days after having a soft/bland diet, they were discharged from the hospital. The degree of pain was assessed by pain score using the Wong-Baker Faces pain rating scale 24 h after surgery^21^.

All patients were followed up regularly using the same protocol at 1, 3, 6, and 12 months, postoperatively. After discharge, routine physical examination, blood tests including nutrition markers (total protein and albumin), and QoL questionnaire surveys were performed in outpatient clinic. In addition, gastroscopy was conducted at 6 and 12 months after surgery. “Readmission” was defined as any admission after discharge within the first 25 postoperative days because of morbidities. Serious adverse event was defined as any unfavourable and unintended experience associated with the intervention, which leads to a life-threatening adverse event, death, persistent or significant disability. “Mortality” was defined as any death that occurred during the hospital stay or any death related to surgery within the first 90 postoperative days.

### Statistical analysis

As described in the protocol, the effective sample size was calculated using short-term complication rates of 7.5% and 13.6% based on reviewing the literature comparing TLDG and LADG for clinical stage I gastric adenocarcinoma^21^.

Two different populations were included in this analysis. The intention-to-treat (ITT) population was defined as all eligible patients who were randomised except those who met the post-randomisation exclusion criteria. The modified per-protocol (mPP) population was the group with pure, intended approaches only, which excluded patients from the ITT population who underwent conversion to open surgery and total gastrectomy. Considering that ITT is used as the main method of analysis in RCTs, evaluation of the main outcome was performed using the ITT population, and the mPP approach was added as a secondary, supportive analysis.

To compare the two groups, Fisher’s exact test and the chi-square test were utilized for categorical variables, whereas Mann–Whitney or Student’s *t*-test test was employed for continuous variables. In addition, multivariate logistic was used to evaluate independent factors affecting morbidity. The difference in QoL trends over time periods between the two groups was analyzed by repeated measure analysis of variance (ANOVA), by controlling preoperative QoL score as the covariate. When the difference in QoL trends over time periods between the groups was detected, analysis of covariance (ANCOVA) was used to compare the QoL at certain postoperative time points (1, 6, 12 months) between two groups. All tests were two-sided, and *P* values less than 0.05 were considered significant. Statistical analyses were performed using the IBM SPSS Statistics software version 25 (IBM Corp.).

## RESULTS

### Patients’ demographics

**Figure 1:**
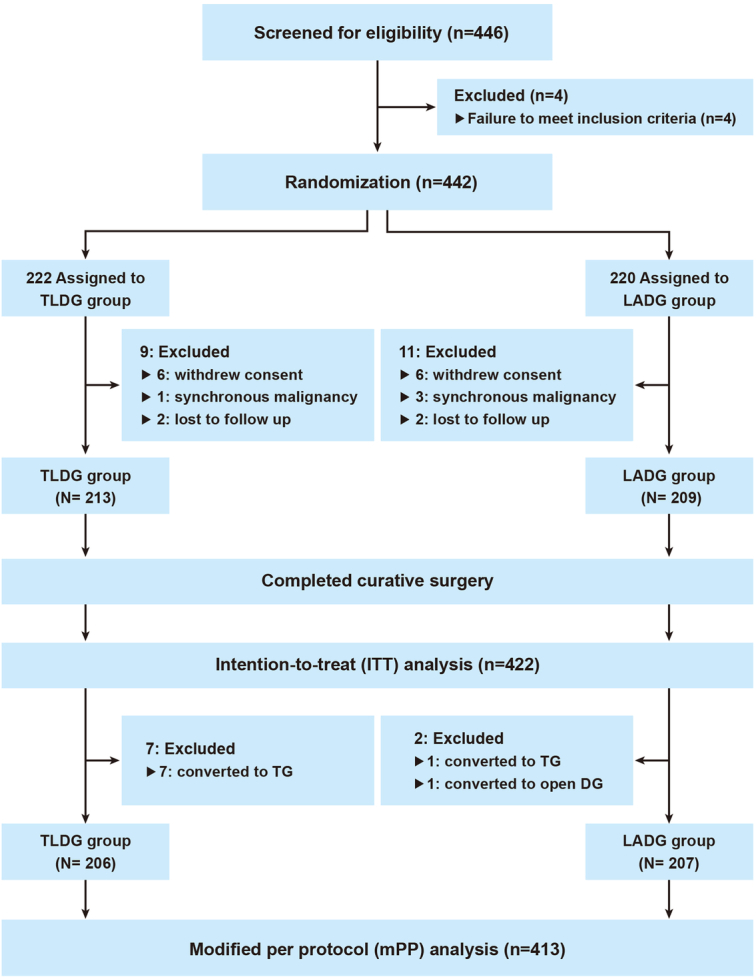
Flow chart of the Korean Laparoendoscopic Gastrointestinal Surgery Study 07 randomized clinical trial. LADG, laparoscopy-assisted distal gastrectomy; TLDG, totally laparoscopic distal gastrectomy.

This trial has completed patient recruiting and enrolment. Figure 1 shows the trial flowchart between January 2018 to September 2020. A total of 442 patients [61.4 (10.5) years; 296 (67.0%) men and 146 (33.0%) women] with clinical stage I gastric adenocarcinoma were enroled and randomly allocated to each treatment group, 222 were randomised to the TLDG group and 220 to the LADG group. After randomisation, 20 patients were excluded for the following reasons 12 withdrew consent, four had synchronous malignancies, and four were lost to follow-up. Finally, 422 patients were included in the ITT population, 213 in the TLDG group and 209 in the LADG group. Among them, eight patients who switched to total gastrectomy and one to open surgery were excluded from the mPP analysis. Thus, the mPP population included 413 patients, with 206 in the TLDG group and 207 in the LADG group.

Patient demographics and baseline characteristics were well balanced between the two groups, regardless of the population being analyzed (Table 1).

**Table 1:** Baseline demographics of the patients.

	Intention-to-treat population			Per-protocol population		
Variables	TLDG (*n*=213)	LADG (*n*=209)	*P*	TLDG (*n*=206)	LADG (*n*=207)	*P*
Age, mean [SD], years	61.4 [10.2]	61.1 [10.4]	0.787	61.5 [10.1]	61.1 [10.4]	0.726
Sex, *n* (%)						
Male	145 (68.1)	135 (64.6)	0.472	140 (68.0)	135 (65.2)	0.602
Female	68 (31.9)	74 (35.4)		66 (32.0)	72 (34.8)	
BMI, mean [SD], kg/m^2^	23.9 [3.1]	24.0 [2.9]	0.901	23.9 [3.0]	24.0 [2.9]	0.856
ASA score, *n* (%)						
I	104 (48.8)	100 (47.8)	0.898	102 (49.5)	100 (48.3)	0.858
II	95 (44.6)	97 (46.4)		90 (43.7)	95 (45.9)	
III	14 (6.6)	12 (5.7)		14 (6.8)	12 (5.8)	
ECOG performance status						
0	184 (84.7)	177 (84.7)	0.679	177 (88.3)	176 (85.0)	0.889
1	29 (13.6)	32 (15.3)		29 (14.1)	31 (15.0)	
Comorbidity	89 (41.8)	83 (39.7)	0.693	86 (41.7)	82 (39.6)	0.689
Hypertension	48 (22.5)	50 (23.9)	0.818	48 (23.3)	50 (24.2)	0.908
Cardiovascular disease	21 (9.9)	23 (11.0)	0.751	20 (9.7)	22 (10.6)	0.871
Pulmonary disease	8 (3.8)	7 (3.3)	0.822	8 (3.9)	7 (3.4)	0.800
Cerebrovascular disease	8 (3.8)	6 (2.9)	0.787	8 (3.9)	6 (2.9)	0.600
Diabetes	19 (8.9)	22 (10.5)	0.624	18 (8.7)	22 (10.6)	0.618
Hepatic disease	3 (1.4)	4 (1.9)	0.722	3 (1.5)	3 (1.4)	0.995
Renal disease	0	3 (1.4)	0.121	0	3 (1.4)	0.248
Other disease	26 (12.2)	21 (10.0)	0.537	25 (12.1)	20 (9.7)	0.435
Previous abdominal operation	38 (17.8)	31 (14.8)	0.432	35 (17.0)	31 (15.0)	0.594
Clinical TNM stage						
cT1N0M0	197 (92.5)	192 (91.9)	0.469	193 (93.7)	191 (92.3)	0.375
cT1N1M0	9 (4.2)	6 (2.9)		8 (3.9)	6 (2.9)	
cT2N0M0	7 (3.3)	11 (5.3)		5 (2.4)	10 (4.8)	
Pathologic T stage						
T1	196 (92.0)	185 (88.5)	0.228	191 (92.7)	184 (88.9)	0.137
T2	10 (4.7)	13 (6.2)		10 (4.9)	13 (6.3)	
T3	6 (2.8)	5 (2.4)		5 (2.4)	5 (2.4)	
T4a	1 (0.5)	6 (2.9)		0 (0)	5 (2.4)	
T4b	0 (0)	0 (0)		0 (0)	0 (0)	
Pathologic N stage						
N0	192 (90.1)	182 (87.1)	0.423	188 (91.3)	181 (87.4)	0.290
N1	10 (4.7)	18 (8.6)		9 (4.4)	18 (8.7)	
N2	8 (3.8)	6 (2.9)		8 (3.9)	6 (2.9)	
N3a	3 (1.4)	3 (1.4)		1 (0.5)	2 (1.0)	
N3b	0 (0)	0 (0)		0	0	
Pathologic stage (AJCC 8th)						
I	198 (93.0)	190 (90.0)	0.689	193 (93.7)	189 (91.3)	0.503
II	13 (6.1)	15 (7.2)		12 (5.8)	15 (7.2)	
III	2 (0.9)	3 (1.4)		1 (0.5)	3 (1.4)	
IV	0 (0)	1 (0.5)		0	0	

TNM stage according to *the Japanese Classification of Gastric Carcinoma*, 4th edition, English.

AJCC, American Joint Committee on Cancer; ASA, American Society of Anesthesiologists; ECOG, Eastern Cooperative Oncology Group; LADG, laparoscopy-assisted distal gastrectomy; TLDG, totally laparoscopic distal gastrectomy.

### Morbidities

**Figure 2:**
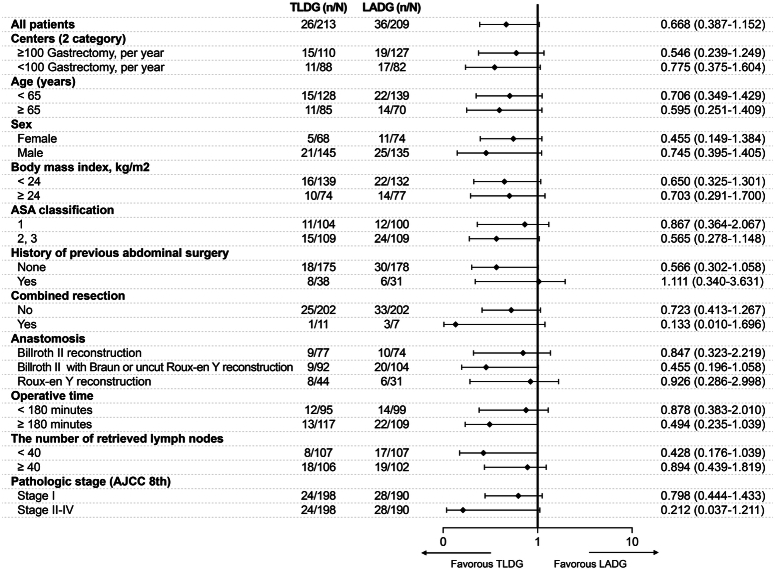
Forest plot of morbidities according to patient subgroups. AJCC, American Joint Committee on Cancer; ASA, American Society of Anesthesiologists; LADG, laparoscopy-assisted distal gastrectomy; TLDG, totally laparoscopic distal gastrectomy.

In ITT analysis, the rate of early complications did not differ between the groups (12.2% vs. 17.2%, *P=*0.169) (Table 2). In a subgroup analysis stratifying patients by baseline characteristics, there was no relationship between the laparoscopic approach and early complication rate (Fig. 2). However, regarding each morbidity subtype, postoperative ileus (POI) (0.9% vs. 5.7%, *P=*0.006) and pulmonary complications (0.9% vs. 4.3%, *P=*0.035) were lower in the TLDG group than that in the LADG group. Other localized and systemic complications and complication grades, according to the Clavien–Dindo classification, were not different between the groups. Finally, the rates of complication greater than or equal to grade II, late complications, readmission within 25 days, and 90 days mortality were similar between the groups. No serious adverse events were observed during the follow-up period. These findings were similarly observed in the mPP analysis (Table 2).

**Table 2:** Early and late postoperative complications.

	Intention-to-treat population, *n* (%)			Per-protocol population, *n* (%)		
Variables	TLDG (*n*=213)	LADG (*n*=209)	*P*	TLDG (*n*=206)	LADG (*n*=207)	*P*
Early complications	26 (12.2)	36 (17.2)	0.169	24 (11.7)	35 (16.9)	0.159
Localized	22 (10.3)	32 (15.3)	0.146	21 (10.2)	31 (15.0)	0.182
Wound	5 (2.3)	4 (1.9)	0.758	5 (2.4)	4 (1.9)	0.751
Fluid collection	6 (2.8)	6 (2.9)	0.973	6 (2.9)	6 (2.9)	0.993
Intra-abdominal bleeding	2 (0.9)	4 (1.9)	0.446	2 (1.0)	4 (1.9)	0.685
Intra-luminal bleeding	1 (0.5)	3 (1.4)	0.369	1 (0.5)	3 (1.4)	0.623
Ileus	2 (0.9)	12 (5.7)	0.006	2 (1.0)	11 (5.3)	0.020
Delayed gastric emptying	3 (1.4)	3 (1.4)	0.981	3 (1.5)	3 (1.4)	0.995
Anastomotic stricture	1 (0.5)	1 (0.5)	0.989	1 (0.5)	1 (0.5)	0.997
Anastomotic leakage	2 (0.9)	3 (1.4)	0.683	1 (0.5)	3 (1.4)	0.623
Pancreatitis/pancreatic leakage	0	1 (0.5)	0.495	0	1 (0.5)	0.318
Systemic	3 (1.4)	8 (3.8)	0.138	2 (1.0)	8 (3.9)	0.105
Pulmonary	2 (0.9)	9 (4.3)	0.035	1 (0.5)	8 (3.9)	0.037
Urinary	0	0	NA	0	0	NA
Renal	0	0	NA	0	0	NA
Hepatic	1 (0.5)	0	0.321	1 (0.5)	0	0.499
Cardiac	0	0	NA	0 (0)	0 (0)	NA
Endocrine	0	0	NA	0	0	NA
Other complication	1 (0.5)	1 (0.5)	0.989	1 (0.5)	1 (0.5)	0.997
Clavien–Dindo complication grade						
I	7 (3.3)	13 (6.2)	0.175	6 (2.9)	13 (6.3)	0.157
II	9 (4.2)	18 (8.6)	0.075	9 (4.4)	17 (8.2)	0.155
IIIa	9 (4.2)	6 (2.9)	0.601	8 (3.9)	6 (2.9)	0.600
IIIb	1 (0.5)	3 (1.4)	0.369	1 (0.5)	3 (1.4)	0.623
IVa	0	3 (1.4)	0.121	0	3 (1.4)	0.248
IVb	0	0	N/A	0	0	NA
V	0	0	N/A	0	0	NA
Late complications	2 (0.9)	6 (2.9)	0.172	2 (1.0)	6 (2.9)	0.284
Intestinal obstruction	1 (0.5)	4 (1.9)	0.212	1 (0.5)	4 (1.9)	0.372
Stenosis	0	0	NA	0	0	NA
Delayed gastric emptying	1 (0.5)	0 (0)	0.321	1 (0.5)	0 (0)	0.499
Reflux symptoms	0	0	NA	0	0	NA
Post gastrectomy symptoms	0	2 (1.0)	0.245	0	2 (1.0)	0.499
Chronic wound complications	0	0	NA	0	0	NA
Readmission	6 (2.8)	10 (4.8)	0.319	5 (2.4)	10 (4.8)	0.293
Serious adverse events	0	0	NA	0	0	NA
90-day mortality	0	0	NA	0	0	NA

“Readmission” was defined as any admission after discharge within the first 25 postoperative day due to morbidities.

“Mortality” was defined as any death occurring during hospital stay or any death related to surgery within the first 90 postoperative days.

LADG, laparoscopy-assisted distal gastrectomy; NA, not applicable; TLDG, totally laparoscopic distal gastrectomy.

Multivariate logistic regression analysis for variables including age, sex, BMI, ASA classification, comorbidities, history of previous abdominal surgery, TLDG (vs. LADG), combined resection, reconstructive methods, operative time, number of retrieved LNs, and pathologic T and N stages revealed that LADG was the only independent factor for the incidence of ileus and pulmonary complications (all *P<*0.05) (eTable 1 and 2 in the Supplement, Supplemental Digital Content 2, http://links.lww.com/JS9/C489).

### Quality of life

**Figure 3:**
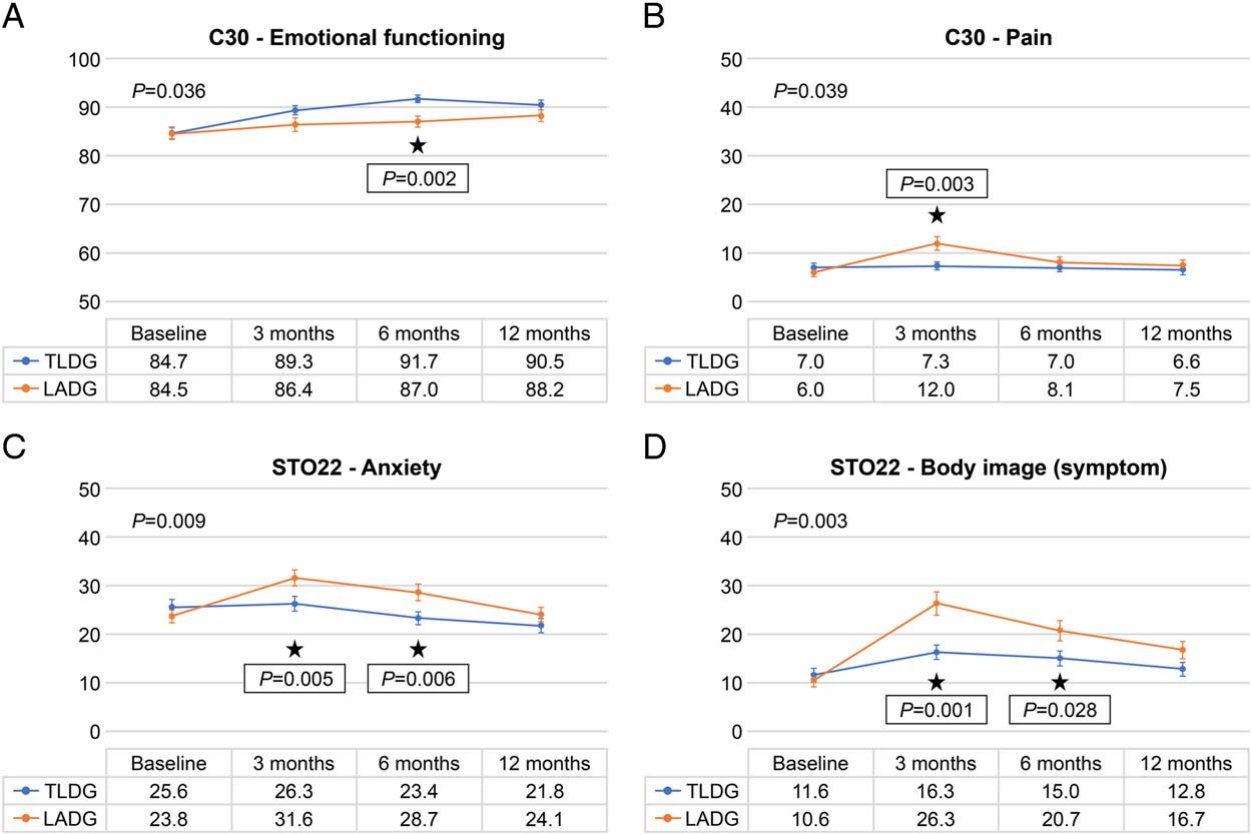
Quality of life (QoL) measurements of the totally laparoscopic distal gastrectomy group (*n*=213) and laparoscopy-assisted distal gastrectomy group (*n*=209) using the Korean version of European Organization for Research and Treatment of Cancer questionnaire. (A) C30 emotional functioning. (B) C30 pain. (C) STO22 anxiety. (D) STO22 body image.

Repeated measure ANOVA revealed that QoL trends during postoperative 1 year were different between the two groups, in terms of C30 emotional functioning (*P=*0.036), C30 pain (*P=*0.039), STO22 anxiety (*P=*0.009), and STO22 body image (*P=*0.003). For those four significant QoL questionnaires, ANCOVA at each three different time points showed that TLDG provided better QoL than LADG, in terms of C30 emotional functioning at 6 months (91. 7 vs. 87.0, *P=*0.002), C30 pain at 3 months (7.3 vs. 12.0, *P=*0.003), STO22 anxiety at 3 and 6 months (26.3 vs. 31.6, *P=*0.005; 23.4 vs. 28.7, *P=*0.006), and STO22 body image at 3 and 6 months (16.3 vs. 26.3, *P=*0.001; 15.0 vs. 20.7, *P=*0.028) (Fig. 3). Other QoL items of EORTC-C30, STO22, questionnaires were not different between the TLDG and LADG groups 1 year postoperatively. (eFigure 1, Supplemental Digital Content 2, http://links.lww.com/JS9/C489)

### Operative and pathologic outcomes

In ITT analysis, TLDG was associated with a significantly shorter length of mini-laparotomy [3.8([0.9) vs. 5.0 (0.9) cm, *P<*0.001] and a lower Wong-Baker Faces pain rating scale [2.7 (1.0) vs. 2.9 (1.0), *P=*0.037]. Operative time, amount of blood loss, the proportion of transfusion, curative (R0) resection, distal gastrectomy, and combined resection did not show any difference between the groups. Anastomotic type, procedures for closing the common entry hole, and anastomotic time did not differ between the groups. The mean number of retrieved lymph nodes (LNs) was similar between the groups [40.2 (15.6) in TLDG vs. 40.9 (16.8) in LADG, *P=*0.672]. The mean tumour size and lengths of the proximal and distal resection margins were similar between the groups. However, seven patients who underwent TLDG and one patient who underwent LADG were converted to total gastrectomy because of positive resection margins (3.3% vs. 0.5%, *P=*0.068). One patient (0.5%) who underwent LADG was converted to open surgery, because of severe adhesions that prevented laparoscopic procedures. Postoperatively, the time to commence the first liquid diet and the length of hospital stay were not different between the groups.

The mPP analysis showed similar results to those of the ITT analysis in terms of operative and pathologic outcomes (eTable 3, Supplemental Digital Content 2, http://links.lww.com/JS9/C489).

### Nutrition and endoscopic findings

The changes in nutritional indicators, including haemoglobin, protein, albumin, and BMI, and the endoscopic findings assessed by residue, gastritis, and bile reflux were not different between the two groups 1 year postoperatively (eFigure 2, Supplemental Digital Content 2, http://links.lww.com/JS9/C489, eTable 4, Supplemental Digital Content 2, http://links.lww.com/JS9/C489).

## Discussion

This trial is the first multi-centre prospective RCT comparing TLDG to LADG for clinical stage I gastric cancer. The present RCT recruited the largest number of patients to date; the RCT did not only focus on short-term morbidities as the primary endpoint but also analyzed QoL, reflecting the impact of treatment from the patients’ perspective. The trial showed that postoperative morbidities did not differ between the two groups. TLDG and LADG equally adopted fine, meticulous lymphatic dissection using a laparoscopic instrument under a magnified camera view, which is known to contribute to the lower incidence of intra-abdominal bleeding and fluid collection in LADG than in open surgery^5^. Moreover, the gap in incisional length between TLDG and LADG was not large enough to cause differences in the rate of wound complications. With sufficient laparoscopic surgical experience, intracorporeal anastomosis is no longer technically demanding, and gastric cancer patients were not extremely obese as in the Western population, enough to cause anastomotic drawbacks^26,27^. Can surgeons gain nothing from TLDG, which pursues minimal invasiveness, by performing complete laparoscopic procedures without an additional incision in the epigastrium?

When analyzed according to each subtype of morbidity, the TLDG could provide less postoperative ileus than the LADG. The pathogenesis of postoperative ileus can be explained as follows: first, increased bowel handling causes gastrointestinal inflammatory response and oedema, leading to ileus^28–30^. Secondly, postoperative pain results in higher catecholamine release and activates the sympathetic nervous system, which inhibits gastrointestinal motility^28,31^. Compared to TLDG, LADG inevitably exerts compressive pressure and excessive traction on gut tissue and disturbs blood flow by holding and stretching it manually when reconstructing gastrointestinal continuity through restricted mini-laparotomy. Moreover, patients in the LADG group had a higher pain rating scale at 24h than those in the TLDG group (eTable 3, Supplemental Digital Content 2, http://links.lww.com/JS9/C489). Our results are in line with the finding that lower abdominal surgery results in less pain than upper abdominal surgery^32^. Interestingly, logistic regression analysis revealed that LADG was the most independent risk factor for postoperative ileus (eTable 1, Supplemental Digital Content 2, http://links.lww.com/JS9/C489). These results may explain why TLDG provided less ileus than LADG. These can be supported by the fact that intracorporeal anastomosis has an earlier day of flatus and less ileus than extracorporeal anastomosis during laparoscopic colectomy^33,34^.

TLDG resulted in fewer pulmonary complications, regarded as the most fatal risk factors for mortality after curative gastrectomy^35,36^. Respiratory function recovery, which is critical for preventing pulmonary complications, can vary according to the incisional location during abdominal surgery. While upper abdominal surgery causes impairment of diaphragmatic function and respiratory muscle dysfunction in 20–40%, the lower one has a normal diaphragmatic function and 2–5% of the prevalence of respiratory muscle dysfunction^37–40^. In contrast, upper abdominal surgery is accompanied by a weakness in diaphragmatic strength and a predominant shifting to rib cage breathing, mainly assisted by the intercostal muscles^40^. Mini-laparotomy of the LADG is located in the epigastrium, and upward movement of the rib cage stretches the peri-incisional skin more in the epigastrium than in the umbilical wound, causing greater pain. Conversely, worse pain from the epigastrium can restrict breathing and intercostal muscle movement more than pain from umbilical wounds; this is accompanied by a decline in respiratory function in the LADG group. Therefore, pulmonary complications can be prevented if the pain is not induced in the upper abdomen between the ribs. This is supported by the fact that intercostal nerve blocks reduce pulmonary complications after upper abdominal surgery^41^. Logistic regression analysis also revealed that TLDG could independently prevent pulmonary complications (eTable 2, Supplemental Digital Content 2, http://links.lww.com/JS9/C489). This is in line with the results of previous studies reporting that pulmonary complications were lower in totally laparoscopic- than in laparoscopy-assisted total gastrectomy^42^.

When comparing surgical procedures with the same morbidities, QoL is an important issue because it reflects the impact of treatment from the patient’s perspective for a considerable time after surgery. Since the TLDG and LADG share similar procedures, most QoL scores were not different within 1 year. However, the intensity of invasiveness perceived by the patients differed according to the type of surgery, and this caused some QoL differences in terms of pain, body image, emotion, and anxiety. Generally, upper abdominal surgery results in higher VAS pain scores than lower abdominal surgery^32^. In this study, LADG caused a greater pain rating scale at 24h than the TLDG (eTable 1, Supplemental Digital Content 2, http://links.lww.com/JS9/C489). Adhesion elicits vague to highly distressing abdominal pain^43^. Since TLDG does not expose the intestine to manual manipulation, it might cause less adhesion-induced pain than LADG, which inevitably requires bowel handling during gastrojejunostomy. Considering that the difference in the improvement of adhesion-induced pain appears 3 months after adhesiolysis, it is convincing that patients who underwent TLDG have less C30 pain at 3 months^44^.

Scars represent the visible sequelae of surgery, and their fading is a symbol of patient recovery. Hypertrophic scar tissue begins to contract between 3 and 6 months after they are formed; at 7 months, its redness subsides, and at 1 year, it fully matures^45–48^. The mini-laparotomy scar within the umbilicus can be hidden in the bottom of the umbilicus and becomes invisible, and the remaining scar beyond the umbilicus is relatively short, which causes high satisfaction of body image in patients that underwent TLDG after 1 year. However, larger scars in the epigastrium would be vividly visible for at least 6 months, which might have caused lower satisfaction with body image in patients that underwent LADG at 3 and 6 months (Fig. 2). Previous studies reported that Scarring causes emotional and psychological suppression^49^. Interestingly, our study also demonstrated that the TLDG was better than the LADG in terms of C30 emotion and STO22 anxiety at 3 and 6 months (Fig. 2). Anxiety is common psychiatric problem and negatively affects the prognosis in gastric cancer patients^50,51^. In addition, the unpredictability of postoperative abdominal pain worsens patients’ emotions and social lives^52^. Therefore, TLDG can be considered for better QoL in terms of pain, body image, emotion, and anxiety at 3 and 6 months after surgery.

Regarding quality control, this trial had more patients with clinical T1N0M0 stage than KLASS-01-RCT (93.0% vs. 77.8%). However, the current trial showed a similar number of retrieved LNs (40.2 in TLDG vs. 40.9 in LADG) to those from KLASS-01-RCT (40.5 in LADG)^4^. In terms of survival prediction, which was not our endpoint, both procedures achieved R0-resection and shared similar laparoscopic procedures, and the overall survival might not be different between the two groups. Besides, the operative time of LADG was shortened by 4 minutes in this trial than in KLASS-01-RCT (180.0 vs. 184.1 min). The operative time of TLDG was similar to that of LADG in KLASS 01 (185.4 vs. 184.1 min)^4^. The conversion rate is another important index for quality control in prospective studies. Only one patient (0.2%) from LADG converted to open surgery, which is far less than previous reports of 0.9–6.4%^2–8^. The qualifications of participating surgeons and quality control seem to be well achieved, as in previous trials.

Our study had several limitations. First, we only included patients with clinical stage I cancer suitable for distal gastrectomy. The use of TLDG for more advanced cancers needs to be clarified in future clinical trials. Second, the results of the trial may not be generalizable to surgeons and centre with less experience. Surgeons who participated in this study were able to finish LADG 4 min faster than those who conducted the KLASS-01-RCT and could perform TLDG at a rate similar to that of LADG without compromising oncological safety. Second, as previously described in the protocol, we excluded BI reconstructions from this study. In BI reconstruction, if a duodenal transection is needed near the superior border of the pancreatic head to leave a sufficient tumour-free margin, LADG, which mainly establishes duodenal transection using an anvil-clamp, can leave a sufficient tumour-free margin, by clamping the part of the duodenum close to the pancreatic border. In the same situation, TLDG, which mainly uses linear stapling for resection and anastomosis, is disadvantageous regarding the anastomosis completion due to the difficulties in making the duodenum redundant, when the duodenal transection staple line is located near the border of the pancreatic head. Although BI-delta can prevent dog-ear creation, it may be performed in a technically tailored manner, and a few surgeons may be reluctant to place the posterior wall of the anastomosis in front of the hepato-duodenal vessels. For these reasons, TLDG (35.1%) adopted fewer BI reconstructions than LADG (66.9%) in South Korea^25^. If the study was performed without excluding BI, the imbalance in BI reconstruction between the TLDG and LADG groups may have acted as a confounder in evaluating the main outcome represented by morbidities. Meanwhile, the use of only GJ-based reconstruction is thought to have shown the incidence of ileus higher in this trial than in other large-scale randomized trials such as KLASS-01, 02 and JCOG0912^3–5^. Finally, the population size in our study was relatively smaller than that in the previous KLASS RCT series. We expect that future studies will consider this in their analyses. Fortunately, a clinical trial (CKLASS-01) with the same sample size and study design was conducted in China at the same time. We plan to collect data from Korea (KLASS-07) and China (CKLASS-01) in consultation with China and analyze them together, yielding a sample size per group of 442 patients and a total sample size of 884 cases.

## Conclusions

TLDG is not different from LADG in postoperative morbidities within 30 days when used for clinical stage I gastric cancer treatment. However, it has benefits in terms of reducing ileus and pulmonary complications. TLDG may provide a better QoL in terms of pain, body image, emotion, and anxiety at 3 or 6 months. The decision to use TLDG or LADG depends on the qualified surgeon with sufficient experience because these QoL differences are usually resolved 1 year after surgery.

## Ethical approval

All procedures were performed in accordance with the ethical standards of the Responsible Committee on Human Experimentation (Institutional and National) and with the Helsinki Declaration of 1964 and later versions. This study was approved by the institutional review board of the Korea University Anam Medical Centre (No. 2017AN0328). The requirement for written consent was waived by the IRB because of the retrospective nature of this study.

## Consent

Written informed consent was obtained from the patient for publication of this case report and accompanying images. A copy of the written consent is available for review by the Editor-in-Chief of this journal on request.

## Sources of funding

This work was supported by the Medtronic, Ltd (ERP 2017-10901). The funders had no role in the design and conduct of the study; collection, management, analysis, and interpretation of the data; preparation, review, or approval of the manuscript; and decision to submit the manuscript for publication.

## Author contribution

S.H.P., and C.M.L. contributed equally to this work and should be considered first coauthors. S.P. had full access to all the data in the study and take responsibility for the integrity of the data and the accuracy of the data analysis, and should be considered corresponding coauthors. Conceptualization: S.-H.P., C.-M.L., S.P. Data curation: all authors. Formal analysis: S.-H.P., C.-M.L., K.-S.Y. Funding acquisition: S.P. Investigation: all authors.

Methodology: S.-H.P., C.-M.L., S.P. Project administration: S.P. Resources: all authors.

Software: all authors.

Supervision: all authors.

Validation: all authors.

Visualization: all authors.

Writing—original draft: S.-H.P., C.-M.L. Writing—review and editing: all authors.

## Conflicts of interest disclosure

S.P. reported receiving grants from Medtronic, Ltd (ERP 2017-10901). All other authors declare that they have no conflicts of interest.

## Research registration unique identifying number (UIN)

1. Name of the registry: https://clinicalTrials.gov.
2. Unique Identifying number or registration ID: NCT03393182.
3. Hyperlink to your specific registration (must be publicly accessible and will be checked): https://clinicaltrials.gov/study/NCT03393182.

## Guarantor

Professor Sungsoo Park is the one who accept full responsibility for the work and/or the conduct of the study, had access to the data, and controlled the decision to publish.

## Data availability statement

The datasets generated and/or analyzed during the current study are not publicly available due to governmental policy regarding individual information but are available from the corresponding author upon reasonable request. The study protocol, statistical analysis plan, and informed consent form are available. Data will only be available for use in meta-analyses of individual participant data, and any request should be submitted with a scientific proposal to the corresponding author. Members of the trial steering committee will evaluate written requests and determine if the request is acceptable. Data will be shared only after a data sharing agreement is fully implemented.

## Supplementary Material
