# Supplementary material for: Totally laparoscopic versus laparoscopy-assisted distal gastrectomy: the KLASS-07: a randomized controlled trial
Source: Int J Surg. 2024 May 6;110(8):4810–20. doi: 10.1097/JS9.0000000000001543 (PMC11325945; doi:10.1097/JS9.0000000000001543)
Supplement: Supplementary file 2 [file js9-110-4810-s002.docx]

**Supplementary Material Online**

**Totally Laparoscopic vs Laparoscopy-Assisted Distal Gastrectomy: The KLASS-07, Randomized Clinical Trial**

**Journal name: *International Journal of Surgery***

**Supplementary Online Content**

**Online tables**

**Supplementary table 1.** Univariate and multivariate logistic regression analyses for variables predicting postoperative ileus.

**Supplementary table 2.** Univariate and multivariate logistic regression analyses for variables predicting postoperative pulmonary disease.

**Supplementary table 3.** Operative and pathological outcomes.

**Supplementary table 4.** Postoperative endoscopic findings in the totally laparoscopic distal gastrectomy group (n = 213) and laparoscopy-assisted distal gastrectomy group (n = 209) at 6 and 12 months.

**Online figures**

**Supplementary Figure 1.** Quality of life (QoL) measurements of the totally laparoscopic distal gastrectomy group (n = 213) and laparoscopy-assisted distal gastrectomy group (n = 209) were performed using the Korean version of the European Organization for Research and Treatment of Cancer questionnaire.

**Supplementary Figure 2.** Nutritional status of hemoglobin, total protein, albumin, and body mass index of the totally laparoscopic distal gastrectomy group (n = 213) and laparoscopy-assisted distal gastrectomy group (n = 209) within 1 year after surgery.

**Supplementary Table 1.** Univariate and multivariate logistic regression analyses for variables predicting postoperative ileus.

| **Variables** | **Morbidity (N, %)** |  | Univariate analysis |  | Multivariate analysis |
| --- | --- | --- | --- | --- | --- |
|  |  | *P* value | Odds ratio (95% CI) | *P* value | Odds ratio (95% CI) |
| **Age** |  |  |  |  |  |
| < 65 | 8 (3.0) |  | 1 |  |  |
| ≥ 65 | 6 (3.9) | 0.630 | 1.304 (0.444-3.829) |  |  |
| **Sex** |  |  |  |  |  |
| Female | 7 (4.9) |  |  |  |  |
| Male | 7 (2.5) | 0.196 | 0.495 (0.170-1.438) |  |  |
| **Body mass index, kg/m^2^** |  |  |  |  |  |
| < 24 | 11 (4.1) |  |  |  |  |
| ≥ 24 | 3 (2.0) | 0.264 | 0.479 (0.132-1.745) |  |  |
| **ASA classification** |  |  |  |  |  |
| < 3 | 13 (3.3) |  | 1 |  |  |
| ≥ 3 | 1 (3.8) | 0.877 | 1.178 (0.148-9.375) |  |  |
| **History of previous abdominal surgery** |  |  |  |  |  |
| None | 12 (3.4) |  | 1 |  |  |
| Yes | 2 (2.9) | 0.832 | 0.848 (0.186-3.877) |  |  |
| **Laparoscopic approach** |  |  |  |  |  |
| Totally laparoscopic | 2 (0.9) |  | 1 |  | 1 |
| Laparoscopy-assisted | 12 (5.7) | 0.016 | 6.426 (1.420-29.076) | 0.018 | 6.915 (1.364-28.132) |
| **Combined resection** |  |  |  |  |  |
| No | 13 (3.2) |  | 1 |  |  |
| Yes | 1 (5.6) | 0.593 | 1.769 (0.219-14.320) |  |  |
| **Reconstructive methods** |  |  |  |  |  |
| Billroth II reconstruction | 4 (2.6) |  | 1 |  |  |
| BII with Braun or uncut Roux-en Y reconstruction | 8 (4.1) | 0.472 | 1.564 (0.462-5.294) |  |  |
| Roux-en Y reconstruction | 2 (2.7) | 0.994 | 1.007 (0.180-5.625) |  |  |
| **Operative time** |  |  |  |  |  |
| < 180 minutes | 8 (4.1) |  | 1 |  |  |
| ≥ 180 minutes | 6 (2.7) | 0.407 | 0.634 (0.216-1.860) |  |  |
| **The number of retrieved lymph nodes** |  |  |  |  |  |
| < 40 | 4 (1.9) |  | 1 |  |  |
| ≥ 40 | 10 (4.8) | 0.104 | 2.652 (0.818-8.592) |  |  |
| **Depth of invasion** |  |  |  |  |  |
| pT1 | 12 (3.1) | 0.560 | 1.577 (0.340-7.304) |  |  |
| pT2-T4 | 2 (4.9) |  |  |  |  |
| **Lymph node metastasis** |  |  |  |  |  |
| pN0 | 11 (2.9) | 0.239 | 2.200 (0.591-8.183) |  |  |
| pN+ | 3 (6.2) |  |  |  |  |

The result was obtained after adjusting the confounding effect of multicenter.

Abbreviations: OR, odds ratio; ASA, American Society of Anesthesiologists; pT, pathological T; pN, pathological N; BII, Billroth II

TNM stage according to *the Japanese Classification of Gastric Carcinoma*, 4^th^ edition, English.

**Supplementary Table 2.** Univariate and multivariate logistic regression analyses for variables predicting postoperative pulmonary complications.

| **Variables** | **Morbidity (N, %)** |  | Univariate analysis |  | Multivariate analysis |
| --- | --- | --- | --- | --- | --- |
|  |  | *P* value | Odds ratio (95% CI) | *P* value | Odds ratio (95% CI) |
| **Age** |  |  |  |  |  |
| < 65 | 8 (3.0) |  | 1 |  |  |
| ≥ 65 | 3 (1.9) | 0.513 | 0.639 (0.167-2.445) |  |  |
| **Sex** |  |  |  |  |  |
| Female | 2 (1.4) |  | 1 |  |  |
| Male | 9 (3.2) | 0.285 | 2.325 (0.496-10.906) |  |  |
| **Body mass index, kg/m^2^** |  |  |  |  |  |
| < 24 | 8 (3.0) |  |  |  |  |
| ≥ 24 | 3 (2.0) | 0.553 | 0.666 (0.174-2.550) |  |  |
| **ASA classification** |  |  |  |  |  |
| < 3 | 10 (2.5) |  | 1 |  |  |
| ≥ 3 | 1 (3.8) | 0.684 | 1.544 (0.190-12.546) |  |  |
| **Previous history of abdominal surgery** |  |  |  |  |  |
| None | 9 (2.5) |  | 1 |  |  |
| Yes | 2 (2.9) | 0.868 | 1.141 (0.241-5.399) |  |  |
| **Laparoscopic approach** |  |  |  |  |  |
| Totally laparoscopic | 2 (0.9) |  | 1 |  | 1 |
| Laparoscopy-assisted | 9 (4.3) | 0.046 | 4.747 (1.013-22.241) | 0.048 | 4.806 (1.017-22.700) |
| **Combined resection** |  |  |  |  |  |
| No | 10 (2.5) |  | 1 |  |  |
| Yes | 1 (5.6) |  | 2.318 (0.280-19.158) |  |  |
| **Reconstructive methods** |  |  |  |  |  |
| Billroth II reconstruction | 2 (1.3) |  | 1 |  |  |
| BII with Braun or uncut Roux-en Y reconstruction | 5 (2.6) | 0.429 | 1.950 (0.373-10.193) |  |  |
| Roux-en Y reconstruction | 4 (5.3) | 0.102 | 4.197 (0.751-23.457) |  |  |
| **Operative time** |  |  |  |  |  |
| < 180 minutes | 5 (2.6) |  | 1 |  |  |
| ≥ 180 minutes | 6 (2.7) | 0.960 | 1.031 (0.310-3.432) |  |  |
| **The number of retrieved lymph nodes** |  |  |  |  |  |
| < 40 | 4 (1.9) |  | 1 |  |  |
| ≥ 40 | 7 (3.4) | 0.342 | 1.828 (0.527-6.341) |  |  |
| **Depth of invasion** |  |  |  |  |  |
| pT1 | 9 (2.4) |  | 1 |  |  |
| pT2-T4 | 2 (4.9) | 0.347 | 2.120 (0.442-10.161) |  |  |
| **Lymph node metastasis** |  |  |  |  |  |
| pN0 | 9 (2.4) |  | 1 |  |  |
| pN+ | 2 (4.2) | 0.477 | 1.763 (0.370-8.413) |  |  |

The result was obtained after adjusting the confounding effect of multicenter.

Abbreviations: OR, odds ratio; ASA, American Society of Anesthesiologists; pT, pathological T; pN, pathological N; BII, Billroth II

TNM stage according to *the Japanese Classification of Gastric Carcinoma*, 4^th^ edition, English.

**Supplementary Table 3.** Operative and pathological outcomes.

| **Variables** | **Intention-to treat Population** | | | | ***P* value** | |  | | **Per protocol population** | | | | | ***P* value** | |
| --- | --- | --- | --- | --- | --- | --- | --- | --- | --- | --- | --- | --- | --- | --- | --- |
|  | **TLDG (n = 213)** | | **LADG (n = 209)** | |  |  |  | | **TLDG (n = 206)** | | | **LADG (n = 207)** | |  |  |
| **Intraoperative outcomes** |  | |  | |  | |  | |  | | |  | |  | |
| Length of mini laparotomy, mean [SD], cm | 3.8 [0.9] | | 5.0 [0.9] | | <0.001 | |  | | 3.8 [0.9] | | | 4.9 [0.8] | | <0.001 | |
| Operative time, mean [SD], minutes | 186.4 [48.0] | | 180.4 [44.3] | | 0.187 | |  | | 185.4 [47.4] | | | 180.0 [44.0] | | 0.231 | |
| Anastomotic time, mean [SD], minutes | 28.4 [14.7] | | 27.8 [12.5] | | 0.673 | |  | | 28.1 [14.3] | | | 27.8 [12.5] | | 0.802 | |
| Blood loss, median [range], mL | 50.0 [0-500.0] | | 50.0 [0-500.0] | | 0.361^*^ | |  | | 50.0 [0-500.0] | | | 50.0 [0-500.0] | | 0.310^*^ | |
| Transfusion | 2 (0.9) | | 1 (0.5) | | 0.576 | |  | | 2 (1.0) | | | 1 (0.5) | | 0.623 | |
| Conversion to open surgery | 0 (0) | | 1 (0.5) | | 0.495 | |  | | 0 (0) | | | 0 (0) | | N/A | |
| Extent of resection |  | |  | |  | |  | |  | | |  | |  | |
| Distal gastrectomy | 206 (96.7) | | 208 (99.5) | | 0.068 | |  | | 206 (100) | | | 207 (100) | | N/A | |
| Total gastrectomy | 7 (3.3) | | 1 (0.5) | |  | |  | | 0 (0) | | | 0 (0) | |  | |
| Combined resection |  | |  | |  | |  | |  | | |  | |  | |
| cholecystectomy | 9 (4.2) | | 6 (2.9) | | 0.639 | |  | | 9 (4.4) | | | 6 (2.9) | | 0.366 | |
| Others | 2 (0.9) | | 1 (0.5) | |  | |  | | 1 (0.5) | | | 1 (0.5) | |  | |
| Anastomosis |  | |  | |  | |  | |  | | |  | |  | |
| Billroth II | 77 (36.2) | | 74 (35.4) | | 0.202 | |  | | 77 (37.4) | | | 74 (35.7) | | 0.338 | |
| Billroth II with Braun | 90 (42.3) | | 98 (46.9) | |  | |  | | 90 (43.7) | | | 98 (47.3) | |  | |
| Roux-en Y | 44 (20.7) | | 31 (14.8) | |  | |  | | 37 (18.0) | | | 29 (14.0) | |  | |
| Uncut Roux-en Y | 2 (0.9) | | 6 (2.9) | |  | |  | | 2 (1.0) | | | 6 (2.9) | |  | |
| **Closure of common entry hole** | | |  | |  | |  | |  | |  |  | |  | |
| Stapling technique | | | 174 (81.7) | | 160 (76.6) | | 0.231 | |  | | 169 (82.0) | 158 (76.3) | | 0.182 | |
| Hand-sewn technique | | | 39 (18.3) | | 49 (23.4) | |  | |  | | 37 (18.0) | 49 (23.7) | |  | |
| Retrieved lymph nodes, mean [SD] | 40.2 [15.6] | | 40.9 [16.8] | | 0.672 | |  | | 40.2 [15.6] | | | 40.8 [16.9] | | 0.682 | |
| Retrieved lymph nodes < 15, mean | 3 (1.4) | | 3 (1.4) | | 0.981 | |  | | 3 (1.5) | | | 3 (1.4) | | 0.995 | |
| Metastatic lymph nodes, mean [SD] | 0.3 [1.2] | | 0.4 [1.5] | | 0.488 | |  | | 0.2 [0.9] | | | 0.3 [1.4] | | 0.326 | |
| Tumor size, mean [SD], cm | 2.9 [2.3] | | 2.7 [2.2] | | 0.439 | |  | | 2.8 [2.3] | | | 2.6 [1.8] | | 0.304 | |
| Length of resected margin |  | |  | |  | |  | |  | | |  | |  | |
| Proximal, mean [SD], centimeter | 5.5 [3.1] | | 5.3 [3.1] | | 0.469 | |  | | 5.5 [3.0] | | | 5.3 [3.0] | | 0.590 | |
| Distal, mean [SD], centimeter | 6.5 [3.3] | | 6.0 [3.2] | | 0.172 | |  | | 6.4 [3.3] | | | 6.0 [3.2] | | 0.277 | |
| R-category |  | |  | |  | |  | |  | | |  | |  | |
| R0 | 213 (100) | | 209 (100) | | N/A | |  | | 206 (100) | | | 207 (100) | | N/A | |
| R1/2 | 0 (0) | | 0 (0) | | N/A | |  | | 0 (0) | | | 0 (0) | | N/A | |
| **Postoperative outcomes** |  | |  | |  | |  | |  | | |  | |  | |
| First diet, mean [SD], day | 3.2 [2.1] | | 3.1 [1.5] | | 0.846 | |  | | 3.2 [2.1] | | | 3.1 [1.5] | | 0.806 | |
| Hospital stays, mean [SD], day | 9.4 [5.2] | | 9.7 [6.3] | | 0.607 | |  | | 9.3 [4.9] | | | 9.7 [6.2] | | 0.491 | |
| Wong-Baker Faces pain rating scale, mean [SD] | 2.7 [1.0] | | 2.9 [1.0] | | 0.037 | |  | | 2.7 [1.0] | | | 2.9 [1.0] | | 0.042 | |

Abbreviations: TLDG, totally laparoscopic distal gastrectomy; LADG, laparoscopy-assisted distal gastrectomy; SD, standard deviation.

**Supplementary Table 4.** Postoperative endoscopic findings in the totally laparoscopic distal gastrectomy group (n = 213) and laparoscopy-assisted distal gastrectomy group (n = 209) at 6 and 12 months.

|  |  | **6 months after surgery** | | | **12 months after surgery** | | |
| --- | --- | --- | --- | --- | --- | --- | --- |
| **Endoscopic finding** | **Grade** | **TLDG group**  **(n = 213)** | **LADG group**  **(n = 209)** | ***P* value** | **TLDG group**  **(n = 213)** | **LADG group**  **(n = 209)** | ***P* value** |
| **Residual food** | 0 | 143 (67.1) | 142 (67.9) | 0.975 | 156 (73.2) | 144 (68.9) | 0.433 |
|  | 1 | 36 (16.9) | 31 (14.8) |  | 33 (15.5) | 36 (17.2) |  |
|  | 2 | 17 (8.0) | 17 (8.1) |  | 13 (6.1) | 15 (7.2) |  |
|  | 3 | 13 (6.1) | 15 (7.2) |  | 9 (4.2) | 14 (6.7) |  |
|  | 4 | 4 (1.9) | 4 (1.9) |  | 2 (0.9) | 0 (0) |  |
| **Gastritis** | 0 | 74 (34.7) | 70 (33.5) | 0.519 | 80 (37.6) | 80 (38.3) | 0.490 |
|  | 1 | 85 (39.9) | 78 (37.3) |  | 72 (33.8) | 76 (36.4) |  |
|  | 2 | 31 (14.6) | 35 (16.7) |  | 42 (19.7) | 33 (15.8) |  |
|  | 3 | 19 (8.9) | 25 (12.0) |  | 15 (7.0) | 19 (9.1) |  |
|  | 4 | 4 (1.9) | 1 (0.5) |  | 4 (1.9) | 1 (0.5) |  |
| **Bile reflux** | 0 | 82 (38.5) | 79 (37.8) | 0.920 | 82 (38.5) | 77 (36.8) | 0.763 |
|  | 1 | 131 (61.5) | 130 (62.2) |  | 131 (61.5) | 132 (63.2) |  |

Abbreviations: TLDG, totally laparoscopic distal gastrectomy; LADG, laparoscopy-assisted distal gastrectomy

**Supplementary Figure 1.** Quality of life (QoL) measurements of the totally laparoscopic distal gastrectomy group (n = 213) and laparoscopy-assisted distal gastrectomy group (n = 209) were performed using the Korean version of the European Organization for Research and Treatment of Cancer questionnaire.

a. Global status

b. Functional scale

c. Symptom scale

**
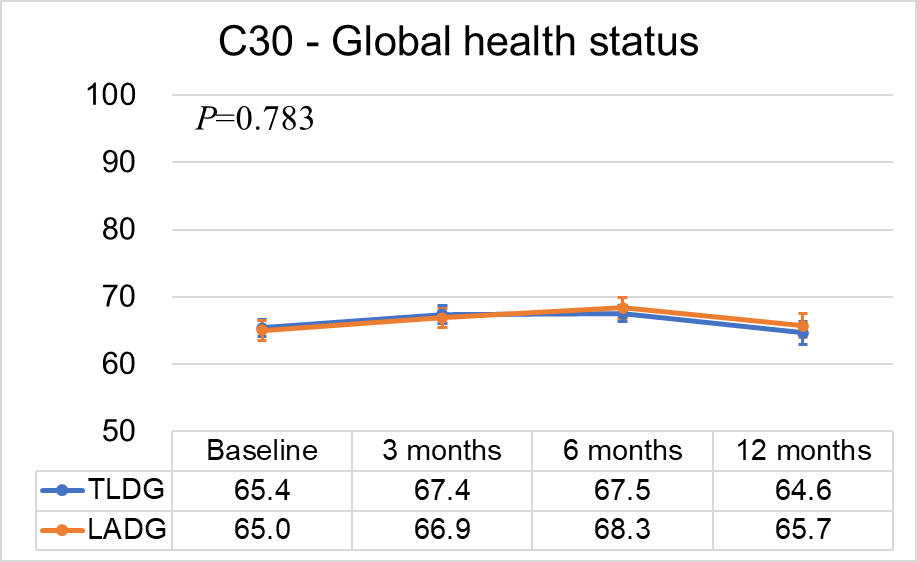
**

**a**

**
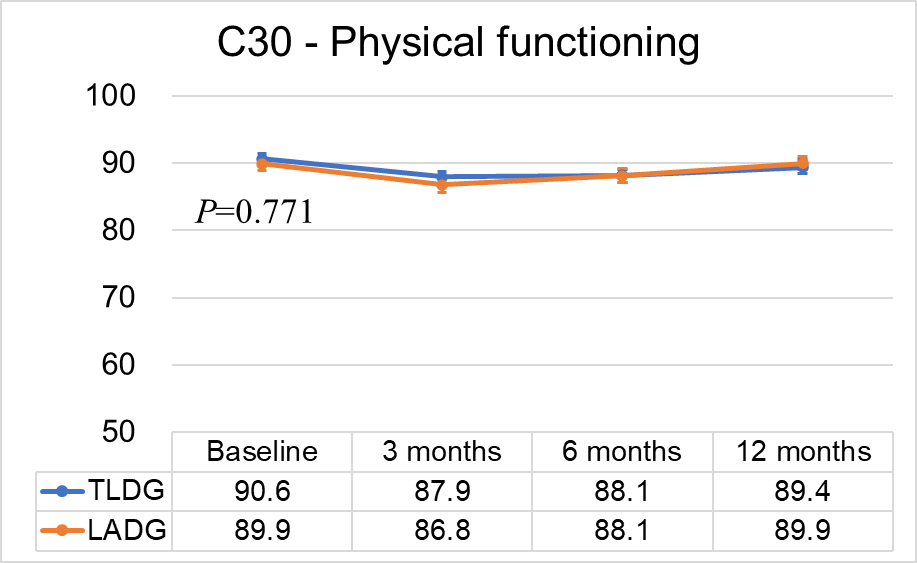

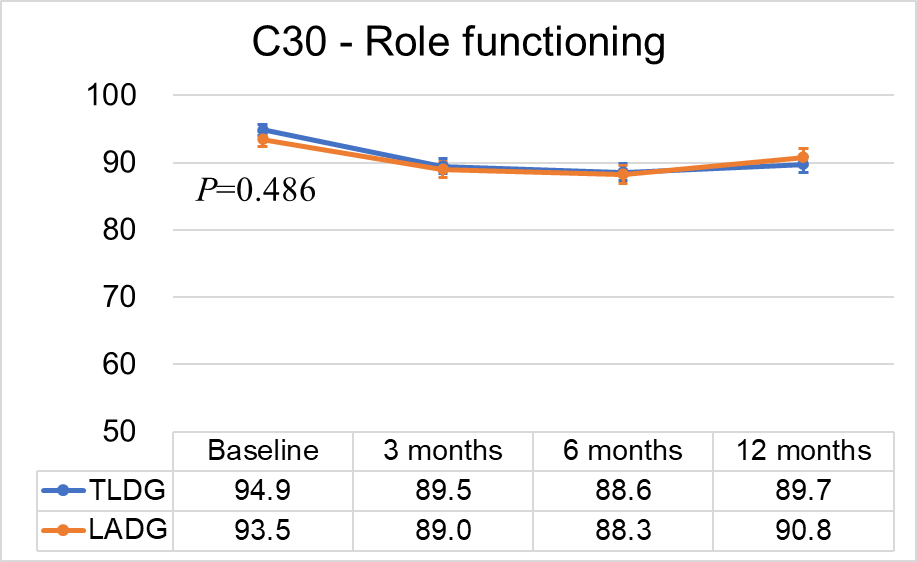
**

**b**

**
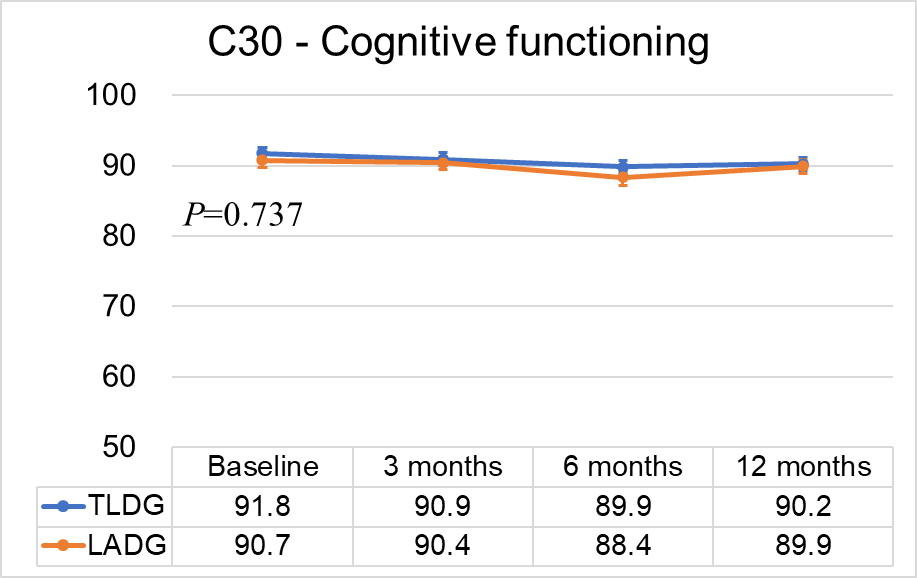

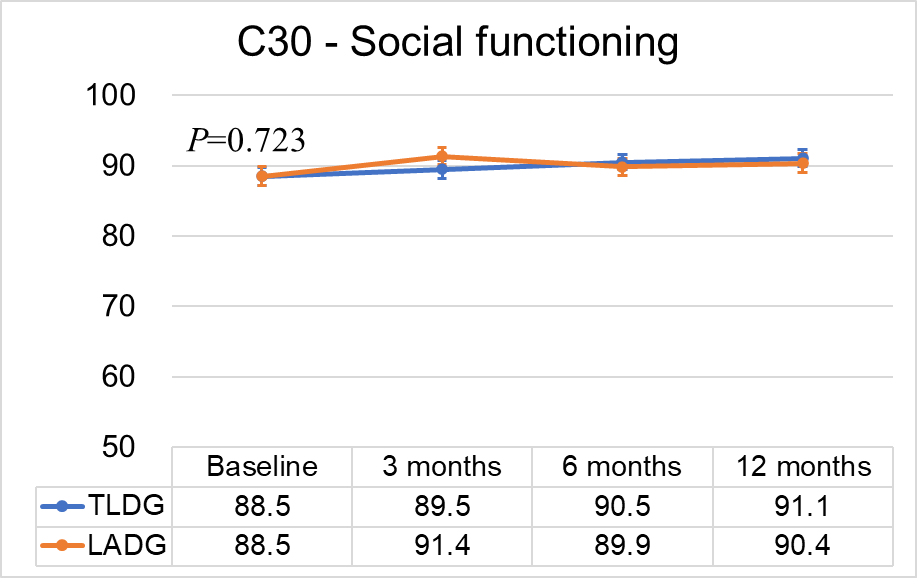
**

**
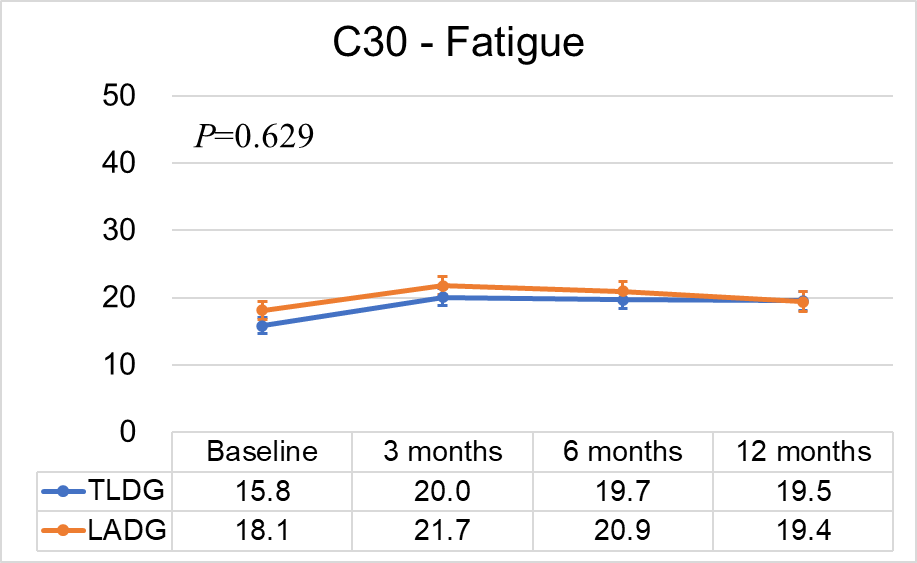

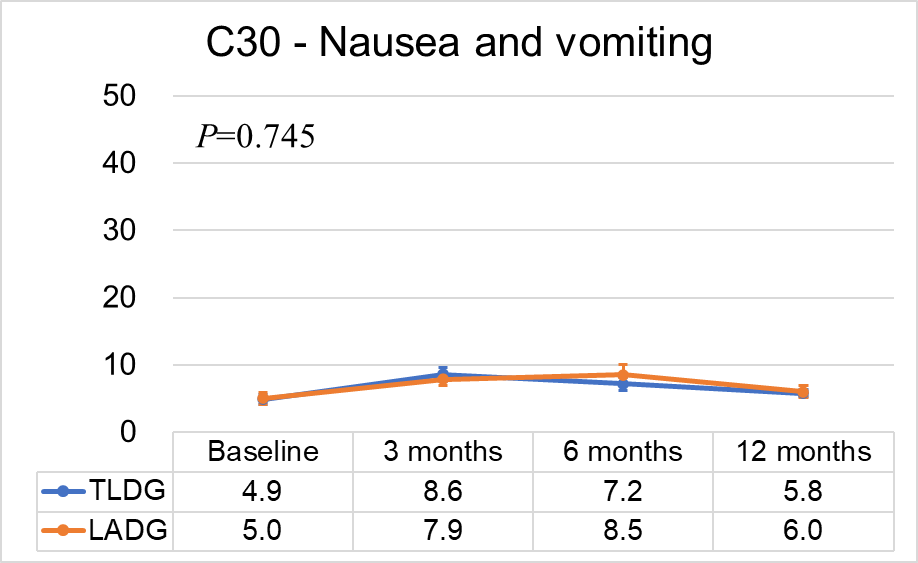
**

**c**

**
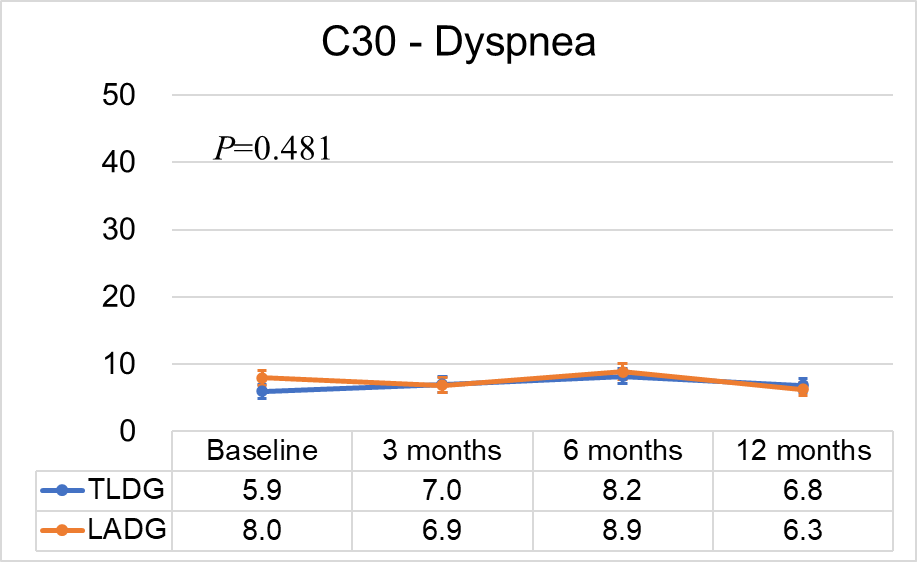

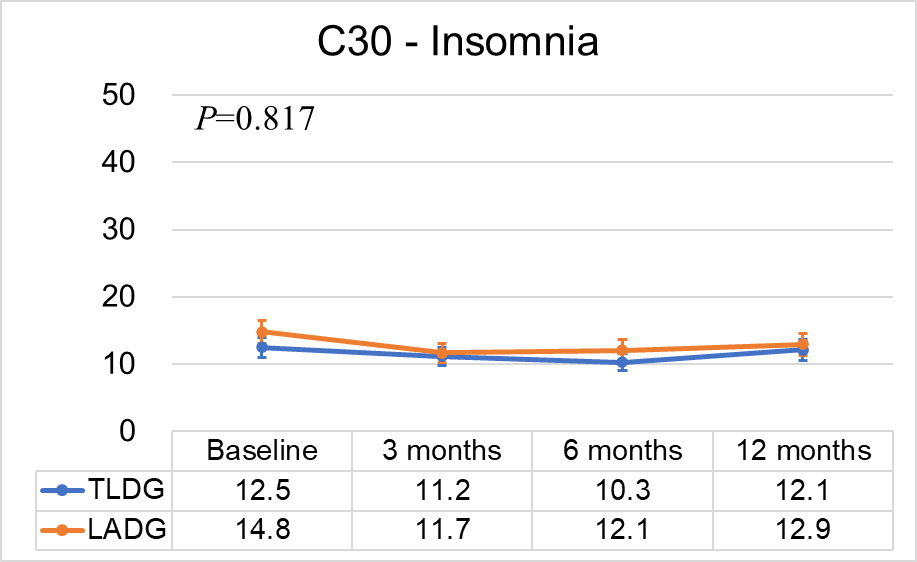
**

**
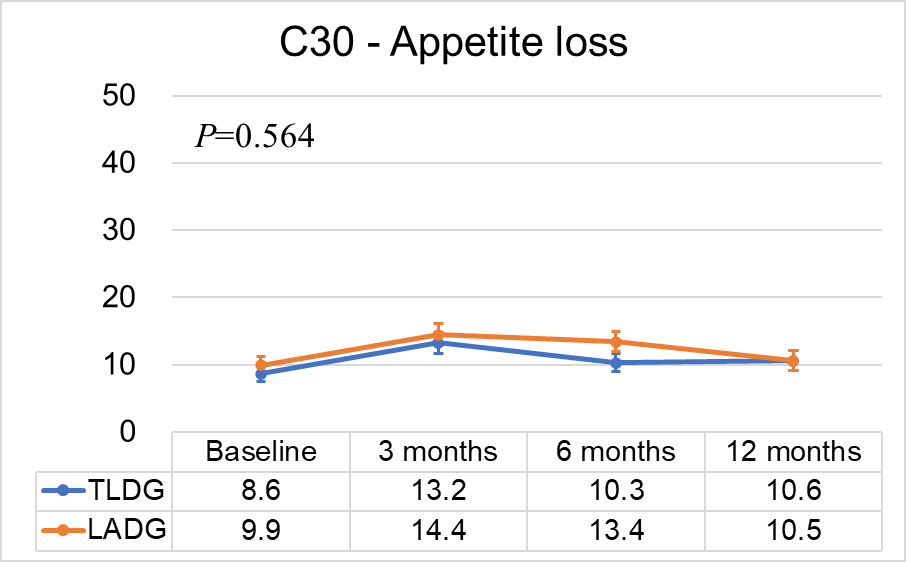

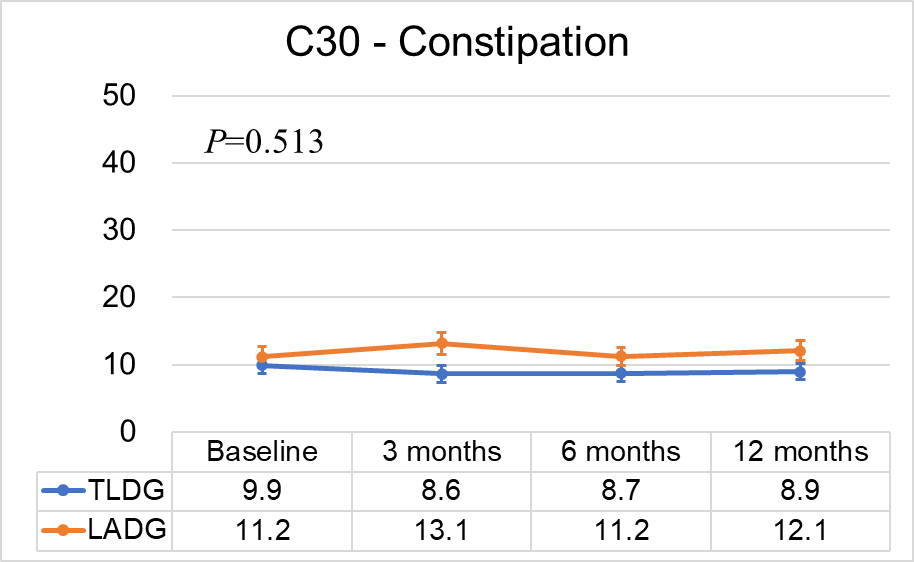
**

**
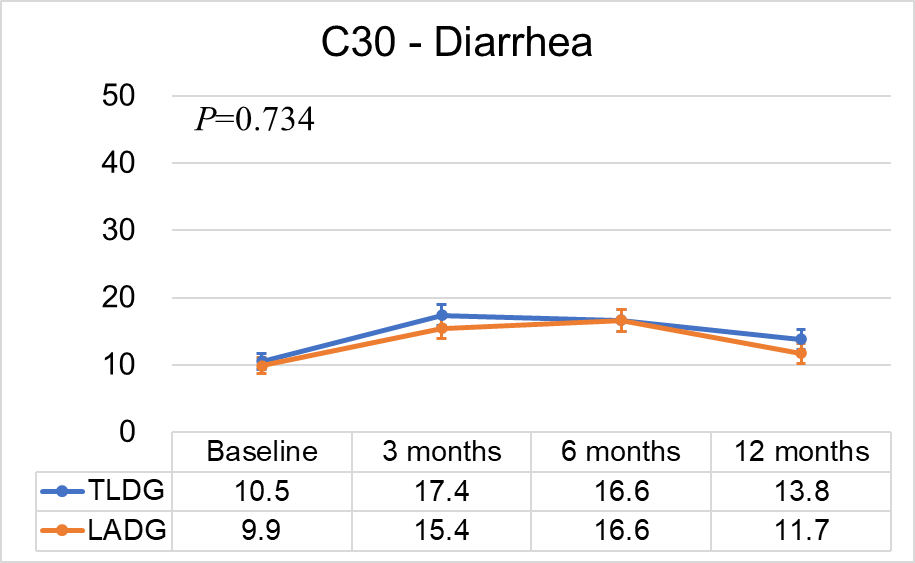

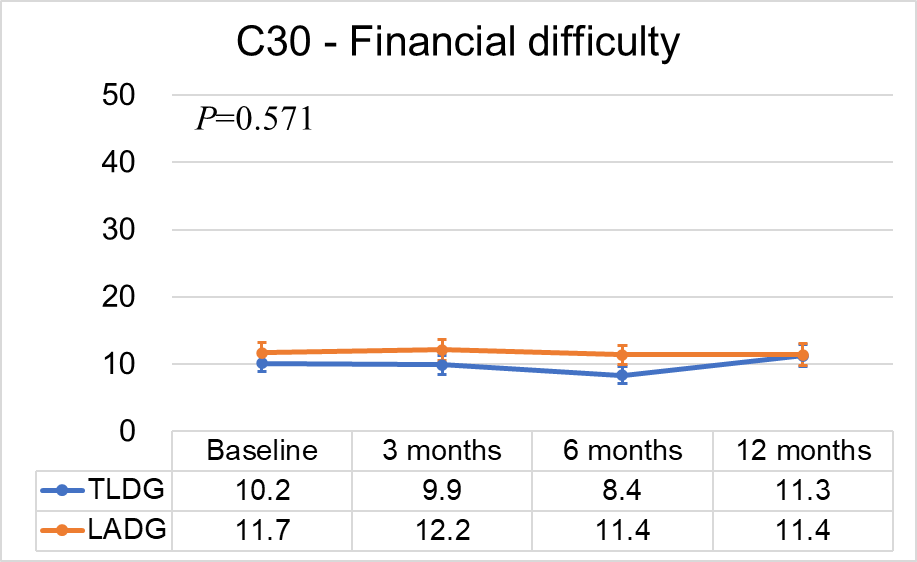
**

**
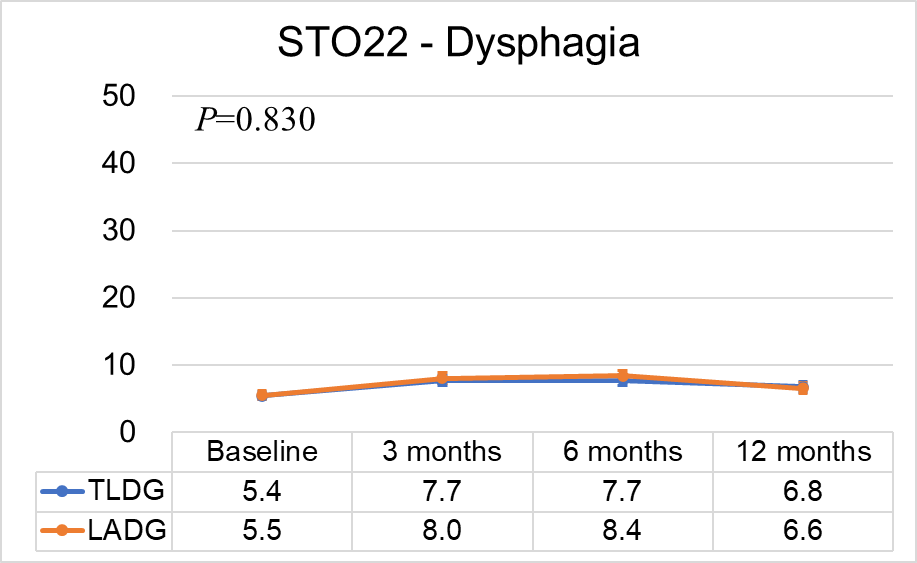

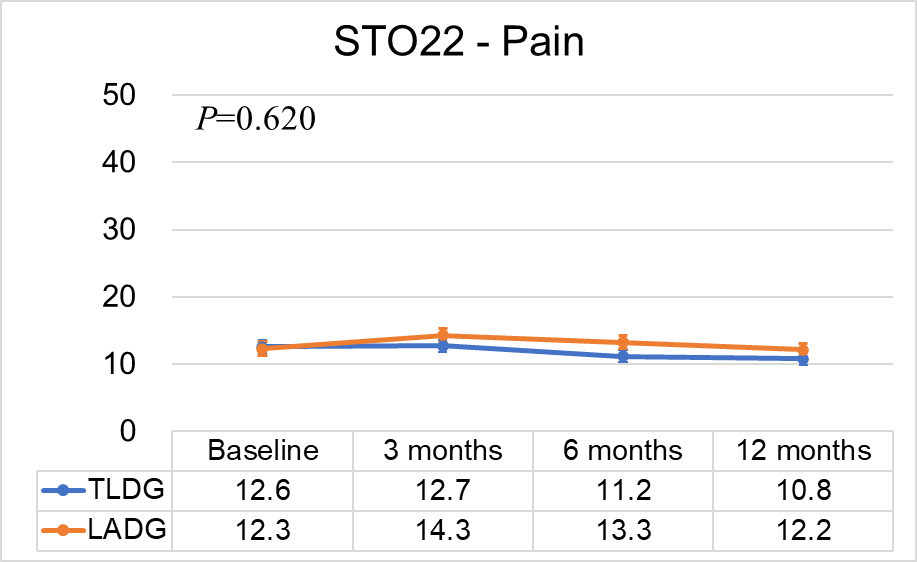
**

**
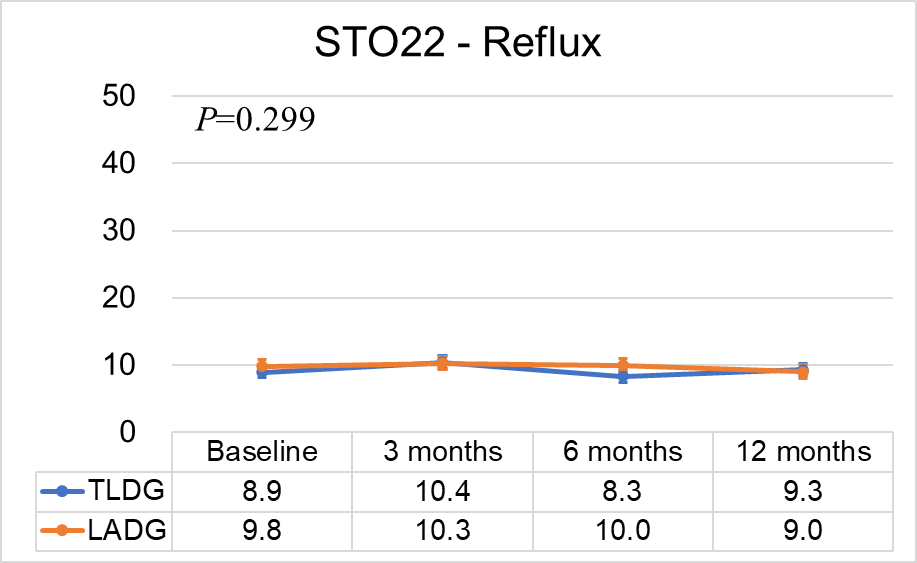

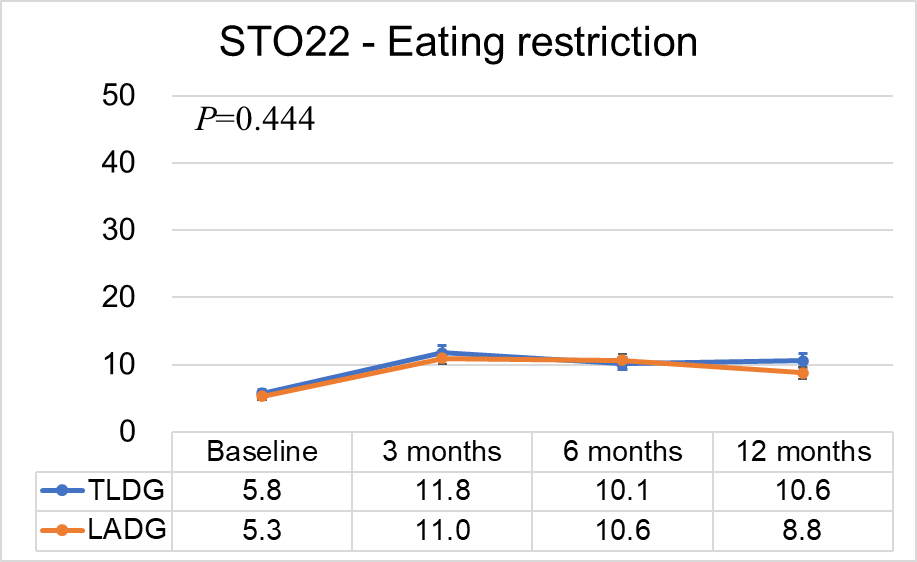
**

**
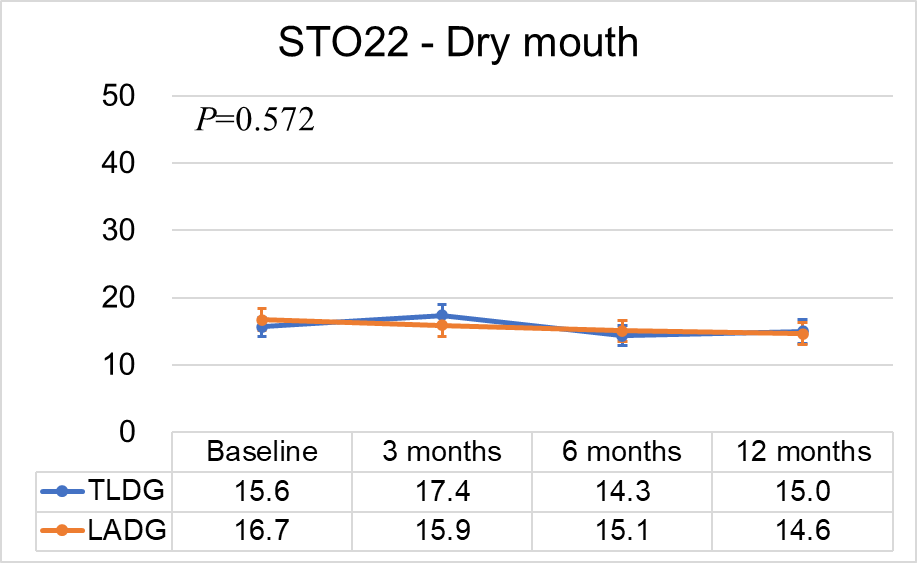

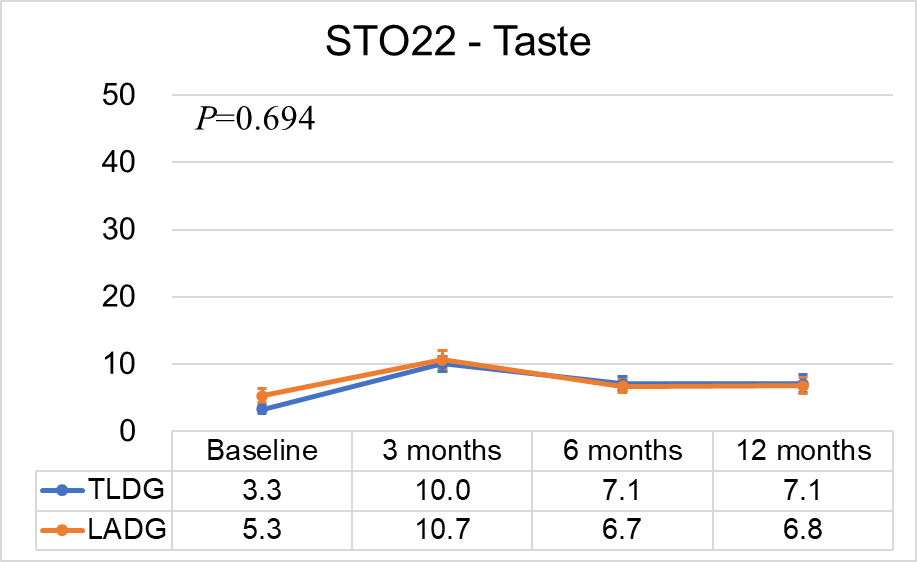
**

**
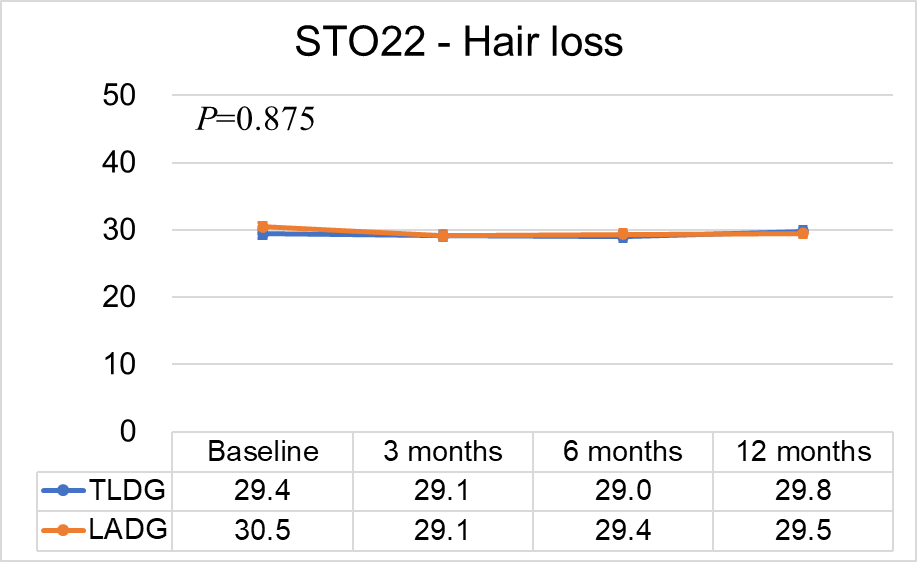
**

**Supplementary Figure 2.** Nutritional status of hemoglobin, total protein, albumin, and body mass index of the totally laparoscopic distal gastrectomy group (n = 213) and laparoscopy-assisted distal gastrectomy group (n = 209) within 1 year after surgery.

a. Hemoglobin

b. Total protein

c. Albumin

d. Body mass index (kg/m^2^)

**
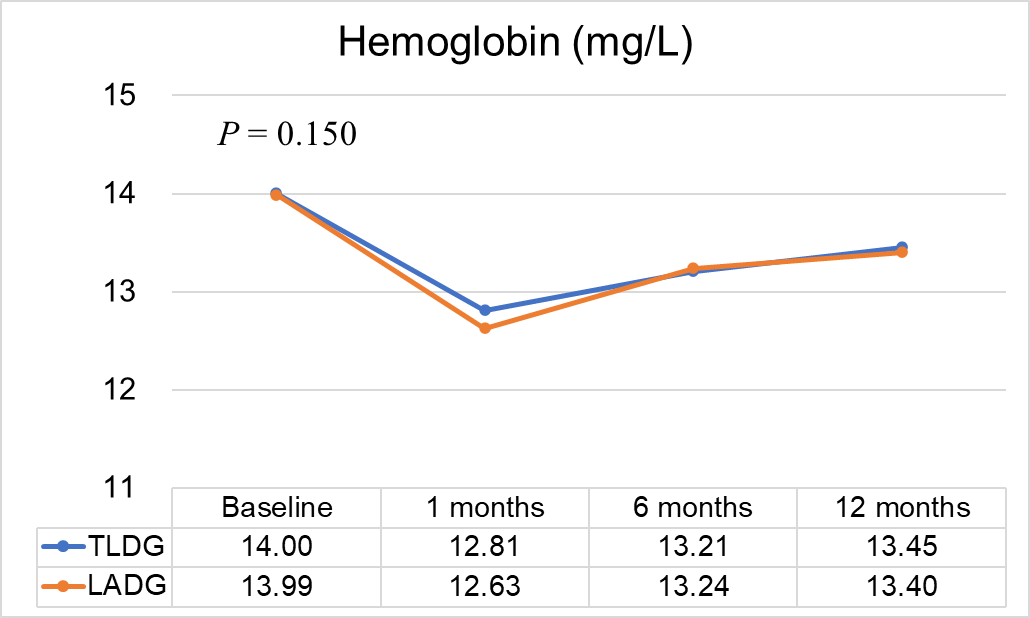

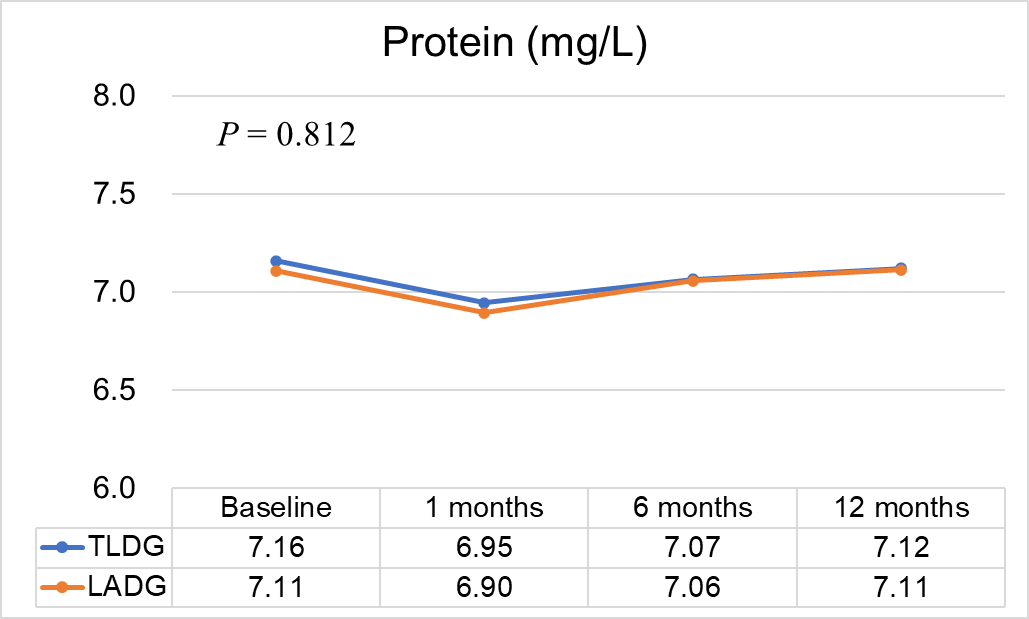
**

**b**

**a**

**
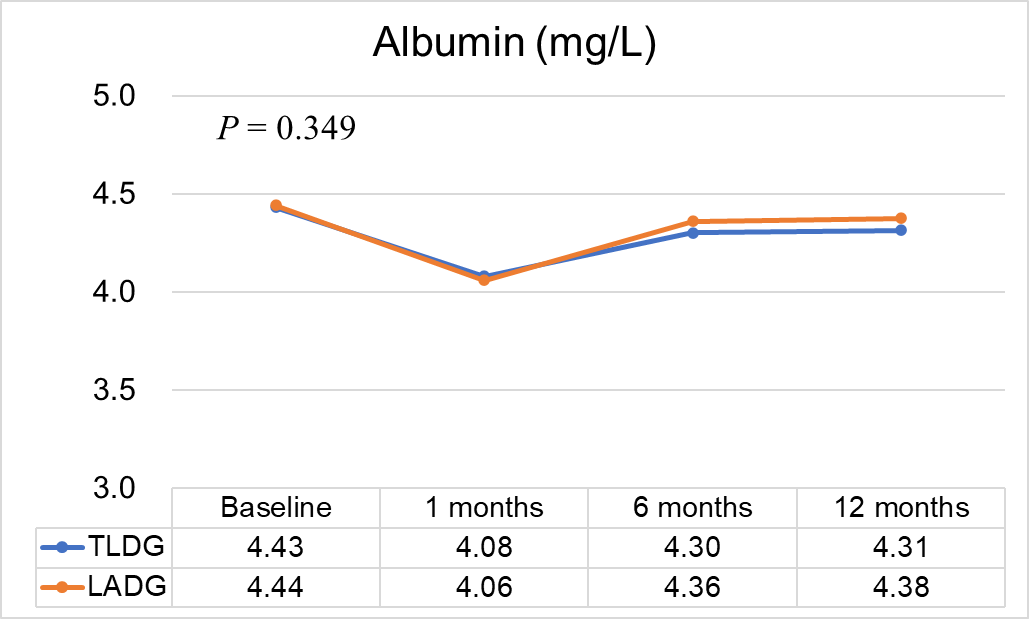

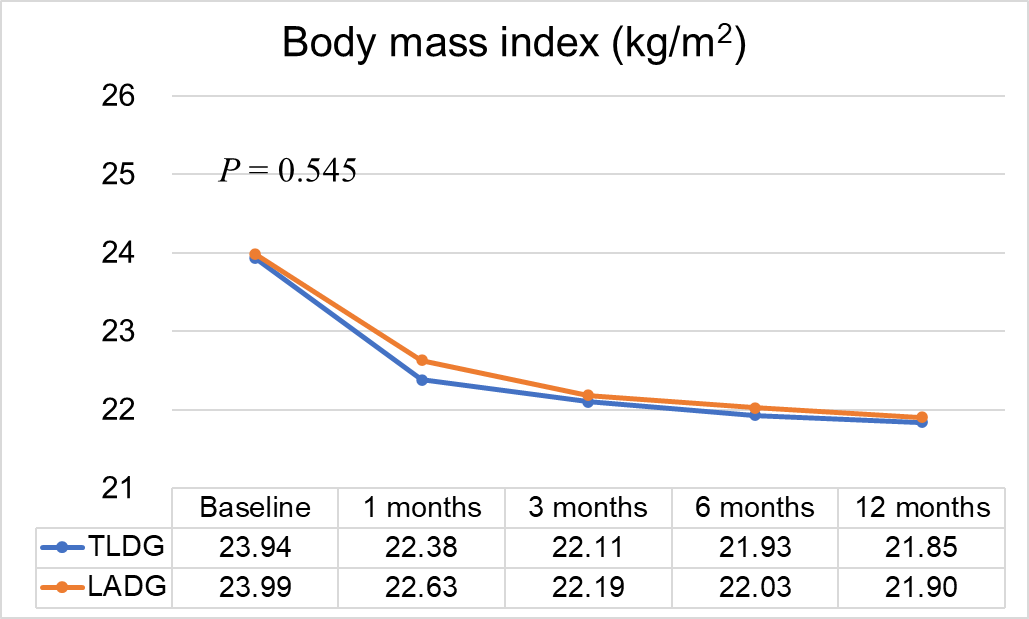
**

**d**

**c**
